# White Light Transmission Spectroscopy for Rapid Quality Control Imperfection Identification in Nanoimprinted Surface-Enhanced Raman Spectroscopy Substrates

**DOI:** 10.1021/acsmeasuresciau.5c00003

**Published:** 2025-03-01

**Authors:** Mike Hardy, Hin On Martin Chu, Serene Pauly, Katie F. Cavanagh, Breandán J.F. Hill, Jason Wiggins, Alina Schilling, Pola Goldberg Oppenheimer, Liam M. Grover, Richard J. Winfield, Jade N. Scott, Matthew D. Doherty, Ryan McCarron, William R. Hendren, Paul Dawson, Robert M. Bowman

**Affiliations:** a Smart Nano NI, Centre for Quantum Materials and Technologies, School of Mathematics and Physics, Queen’s University Belfast, Belfast BT7 1NN, United Kingdom; b Advanced Nano-Materials Structures and Applications Laboratories, School of Chemical Engineering, University of Birmingham, Birmingham B15 2TT, United Kingdom; c Healthcare Technologies Institute, Institute of Translational Medicine, Mindelsohn Way, Birmingham B15 2TH, United Kingdom; d Photonic Integration and Advanced Data Storage CDT, James Watt School of Engineering, University of Glasgow, Glasgow G12 8QQ, United Kingdom; e Yelo Ltd, 20 Meadowbank Rd, Carrickfergus , Co. Antrim BT38 8YF, United Kingdom; f Causeway Sensors Ltd., 63 University Rd, Belfast BT7 1NF, United Kingdom; g Tyndall Micro Nano Electronics, Tyndall National Institute, University College Cork, Lee Maltings, Cork T12 R5CP, Ireland; h Centre for Nanostructured Media, School of Mathematics and Physics, Queen’s University Belfast, Belfast BT7 1NN, United Kingdom

**Keywords:** nanoimprint lithography, surface-enhanced raman spectroscopy, biosensing, white light transmission, custom-built optics, machine learning, nanothermal measurements

## Abstract

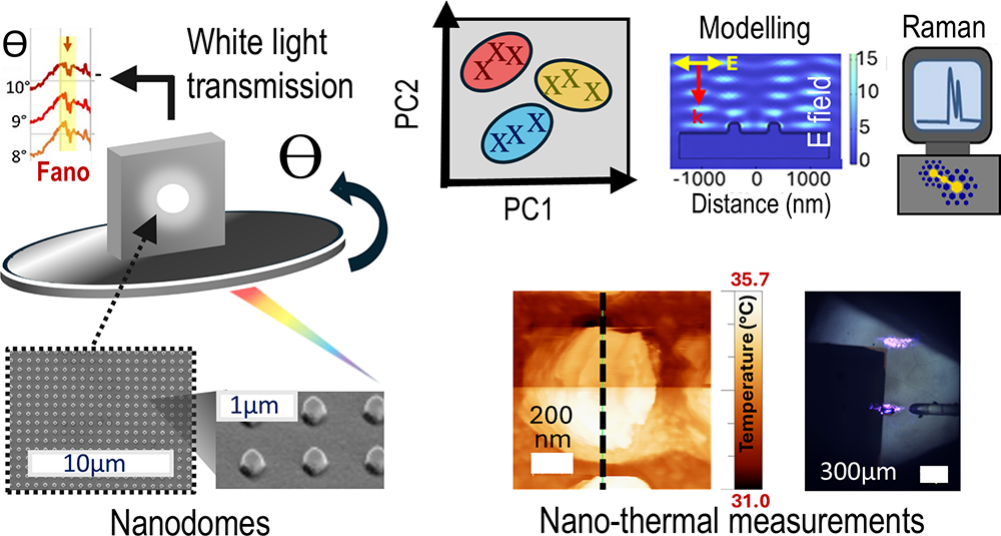

Miniaturized biomedical sensor development requires improvements in lithographic processes in terms of cost and scalability. Of particular promise is nanoimprint lithography (NIL), but this can suffer from a lack of high-fidelity pattern reproducibility between master and imprinted substrates. Herein, we present a multidisciplinary investigation into gold- and iron-coated NIL sensors including custom optics and spectroscopy, scanning probe microscopy, and data analysis insights. Polyurethane NIL-made nanodome arrays were interrogated with white light transmission spectroscopy, coupled with principal component analysis (PCA) to investigate potential offsets in the photon-substrate plane interaction angle, an imperfection in NIL substrates. Large-angle mismatches (2–10°) were found to be easily discernible by PCA with statistically significant differences (*p* = 0.05). Unexpected dips in some spectra are postulated to be due to interacting localized and propagating plasmon polaritons, which is supported with a coupled two-oscillator model. General insights are made regarding the interpretation of PCA loadings, which should be related to physical phenomena, and where maximum variance is not necessarily the most meaningful criterion. Smaller angles (<1°) show no significant differences with overlapping confidence intervals in PCA space. Surface-enhanced Raman spectroscopy (SERS) measurements on gold-coated nanodomes returned relative standard deviations of 6–10% via analysis of gelatin, which is of interest as a nasal lining approximation. Interestingly, nanodomes coated in iron produced small, but useful SERS enhancements, which was subsequently interrogated via scanning thermal probe microscopy showing temperature increases of up to 5 °C over the area of one nanostructure (∼1 μm^2^). Nanostructures remained intact despite the surprising large local temperature increase relative to a gold-coated comparison sample (∼2 °C). The current study provides a framework for the rapid and accurate quality control assessment of imperfections in NIL-produced nanostructures for sensing applications in SERS and surface plasmon resonance, which may need precisely fabricated nanostructures.

## 1 Introduction

Miniaturized sensing continues to expand with applications in environmental monitoring,^1,2^ food safety,^3−5^ defense and security,^6−8^ and an array of healthcare spheres.^9−12^ The challenge, however, is maintaining suitably high performance, in terms of analytical sensitivity and selectivity (specificity) as components are miniaturized, which has led to device comparison studies.^11,13,14^ Alongside this is a need for a reduction in costs; portable sensing devices, including “smart wearables”, need to be inexpensive and may even be single-use disposables, and thus requiring production in large volumes. A good recent example are the SARS-COVID19 lateral flow testing kits, which were supplied *en masse* to many populations during the coronavirus pandemic to control virus spread. The tests’ material cost was as low as $1, critical when mass testing is being performed,^15,16^ but had comparatively low analytical sensitivity, only returning a positive test result for high viral loads.

A prominent class of sensors is plasmonics-based platforms, those that make use of interfacial surface-bound collective electron-photon hybrid excitations (plasmon-polaritons),^17,18^ which can concentrate electromagnetic fields around nanostructures coated with certain metals, typically gold and silver in the visible range for optimal EM field enhancements.^19^ These sensors can be further divided into two classes. First, surface plasmon resonance (SPR) sensors that monitor the spectral resonance position change because of the introduction of a proximal medium of interest, e.g., gas or liquid flow with variable refractive index. The second class of plasmonic sensor is surface-enhanced Raman scattering/spectroscopy (SERS) sensors, where the large local electric fields are used to amplify Raman scattering—the inelastic scattering of light—from the vibrational bonds in nearby molecules.^20^

SERS is a rapidly expanding and evolving area and nowadays explores a variety of nanoparticle shapes often fabricated by bottom-up methods.^21^ However, quantification is still an ongoing concern^22−24^ and thus, control of feature sizes on the nanoscale has been desirable for more uniform SERS signals across individual SERS substrates and across substrate batches for analytical uses, i.e., precise quantification of various molecules, and has subsequently led to the development of a range of more ordered top-down fabricated SERS nanogeometries. Recently, SERS has appeared in a variety of lateral flow immunoassays, analogous to COVID tests, promising increasing sensitivity, multiplexing capability, and quantitative determination.^25−27^

**Figure 1:**
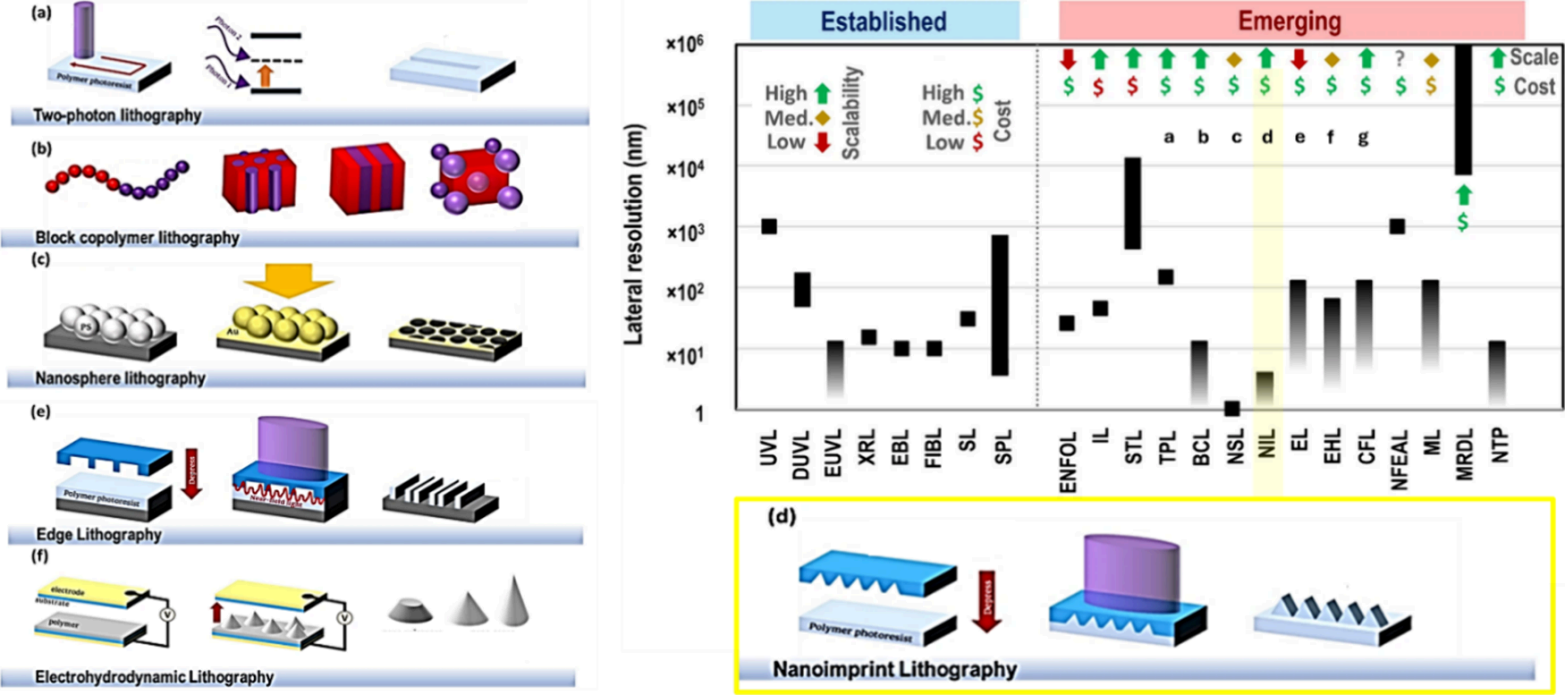
Emerging lithographic techniques. Schematics of emerging lithographies. (a) Two-photon lithography, (b) block copolymer lithography, (c) nanosphere lithography, (d) nanoimprint lithography (bottom R), (e) edge lithography, and (f) electrohydrodynamic lithography. Lithographic resolutions and assessment of scalabilities and costs in summary graph (TOP R) – UVL = conventional UV lithography, DUVL = deep ultraviolet lithography, EUVL = extreme ultraviolet lithography, XRL = X-ray lithography, EBL = electron beam lithography, FIBL = focused ion beam lithography, SL = soft lithography, SPL = scanning probe lithography, ENFOL = evanescent near-field optical lithography, IL = immersion lithography, STL = stereolithography, TPL = two-photon lithography, BCL = block copolymer lithography, NSL = nanosphere lithography, NIL = nanoimprint lithography, EL = edge lithography, EHL = electrohydrodynamic lithography, CFL = capillary force lithography,, NFEAL = near-field electrospinning-assisted lithography, ML = magnetolithography, MRDL = magnetorheological drawing lithography, NTP = nanotransfer printing. Full details of all techniques in Stokes et al. Reprinted or adapted with permission under a Creative Commons CC-BY-4.0 license from ref (30). Copyright 2023 Stokes, K. et al., Springer Publishing.

Top-down SERS substrates are therefore of a different character^28^ to earlier generations of SERS media, require different performance characterization strategies,^29^ and sacrifice a degree of sensitivity for better uniformity/reproducibility. Electron beam methods have been used for precise nanopatterning, which can also provide intricate shapes. However, such techniques are often time-consuming and costly, and therefore not amenable to mass production for real-world adoption. This is a nanofabrication sticking point at large, and research into emerging lithographies, which balance precise nanometric feature size creation with reproducibility, costs, and scalability, is ongoing.^30^ Of particular promise is nanoimprint lithography (NIL), which uses an initial e-beam made master template to stamp a pattern, repeatedly, into batches of polymer forming an inverse replica of the template.^31^ The technique thus offers a pathway to repeatable nanometrically sized patterns, at suitable throughput and cost, including SERS substrate development. Stokes et al. have identified NIL as an emerging candidate amenable to scalability, high volume manufacturing, and low cost^30^ (Figure 1). Furthermore, the technique achieves little waste.

NIL has already found industry adoption. Notably, Canon Inc. (USA) has stated plans to use NIL in production of NAND flash memory devices, as in solid-state and USB pen drives, with a projection of process cost reduction of up to 40%.^32^ Moreover, recently NIL Technology (Denmark), which provides nanostructure-based meta-optics, has invested 31 M USD in upscaling its NIL production processes, projecting application to smartphone, augmented/virtual reality (AR/VR) headsets, and robotic technologies.^33^ However, fabrication imperfections still exist, especially away from large-scale company operation, arising, for instance, from incomplete depression of the template into the polymer base, or dust or other particles getting trapped in the mold. While lagging behind the low defect rate associated with photolithography, NIL in general performs well in terms of defect rate compared with electron beam or focused ion beam techniques.

Another specific problem in NIL fabrication is depression angle mismatch. This is where the template is not pressed at a perfect 90° perpendicular angle with the polymer plane, usually because the polymer surface is uneven or nonplanar.^34^ Subsequently, a laser beam hitting the resulting SERS substrate surface will inevitably excite different plasmonic modes, essentially “seeing” a different geometric structure. Such a discrepancy is difficult to detect visually and may require time-consuming scanning probe microscopy or substrate cross-sectioning as quality control. It is therefore imperative that fast and inexpensive methods are sought for the assessment of possible defects in NIL platforms.

In this study, we investigate the possibility of using white light spectroscopy, but with an optimal (transmission) optical setup and novel data analysis strategy to judge depression angle mismatch in a periodic nanodome plasmonic SERS sensor. As a sensing application, we present measurements on gelatin, which approximate the mucosal lining environment for a SARS-COVID19 nasal spray. The investigation discusses three interesting findings besides the main goals: (1) the origin of asymmetric (Fano) resonances in the high-angle white light transmission spectra (and insight into plasmonic resonance spectral positions), (2) on the interpretation of principal component analysis data sets where the principal component of maximum variance is not necessarily most meaningful, and (3) on the use of alternative plasmonic materials, which can be useful despite conferring only modest enhancements to the Raman signal. Our study thus follows the following pathway:

1A. White light transmission spectra as a function of light incident angle
1B. Spectral analysis: relation of experimental spectra to asymmetric (Fano) resonances
1C. Spectral analysis: commentary on interpretation of principal component analysis (PCA) data
2A. Surface enhanced Raman spectroscopy (SERS) measurements on gold–iron nanodome samples
2B. SERS measurements on bare iron nanodome samples, with commentary on alternative plasmonic materials and thermal properties of substrate studied (via scanning thermal probe microscopy)

## 2 Experimental Section

### 2.1 Nanostructure Fabrication

Nanodome samples were fabricated via soft NIL analogous to ref (35), which has studied periodic nanodome sensors in an SPR context. A layer of photoresist coating on a silicon wafer was patterned by e-beam (EULITHA AG, Switzerland) before a reactive ion etch was used to pattern the nanodome structures into the silicon (master template). Then, a two-step polydimethylsiloxane (PDMS) application was applied,^36^ first hard PDMS spun at 500 rpm for 5 min and then soft PDMS (Sylgard 184 kit) and a cure at 60 °C (140 °F) (submaster template). Photocurable polyurethane (PU) (Norland Optical Adhesive 63, Norland Products, New Jersey, USA) was then dropped onto a glass slide and the submaster template firmly depressed in. To harden the structures, 365 nm UV light was applied for 10 min (26 mW/cm^2^).^35^ The structure dimensions on the submaster template were 200 nm × 200 nm in *x* and *y* (dome sides) and 200 nm in depth/height, i.e., 200 nm-sided cube. There is some relaxation of the PU to form a more dome-like shape. A period of 800 nm (dome-to-dome, center-to-center) was specified for the array; measurements reveal that this is closer to 790 nm (please see the Supporting Information for further details on dome shape and periodicity). The SERS active area is 5 mm × 5 mm.

### 2.2 Nanostructure Metallization

Nanodome samples were evaporated with iron (30 nm) in a thermal deposition system (Moorfield Nanotechnology, UK) at high vacuum (<1 × 10^–5^ mbar) in a titanium basket, monitored via a quartz crystal microbalance sensor. Samples were evaporated in triplicate, “A”, “B”, and “C”. Nanodome sample C was selected for white light and Raman spectroscopy measurements. Nanodome sample A was used for subsequent thermal probe microscopy analysis (see below), a modified atomic force microscope with light illumination on the top (fiber optic) or bottom surface, and thus able to record local temperature (Attocube Systems AG, Germany). Film composition was verified by energy-dispersive X-ray spectroscopy/spectrometry at 10 keV (Figure S5) using a separate calibration glass slide metallized alongside the nanodome samples. Prior to metallization, the glass slide was cleaned vigorously with electronic brush, flowing water, and isopropanol application. Subsequent gold metallization (40 nm) on the iron base layer for SERS measurements was carried out via an automatic sputtering system (Agar Scientific Ltd., UK). The gold films produced for the comparison scanning thermal probe microscopy (SThM) measurements (see below) were metallized separately (details in the Section S5).

### 2.3 Nanodome Imaging

Scanning electron microscopy (SEM) images were acquired in a JEOL SEM (Japan). Optical images of nanodome samples were taken with a digital microscope (VHX Series digital microscope, Keyence Co., UK) and nanostructure period measured with ImageJ (W.S. Rasband, National Institutes of Health, USA) software.^37^

### 2.4 White Light Spectroscopy

**Figure 2:**
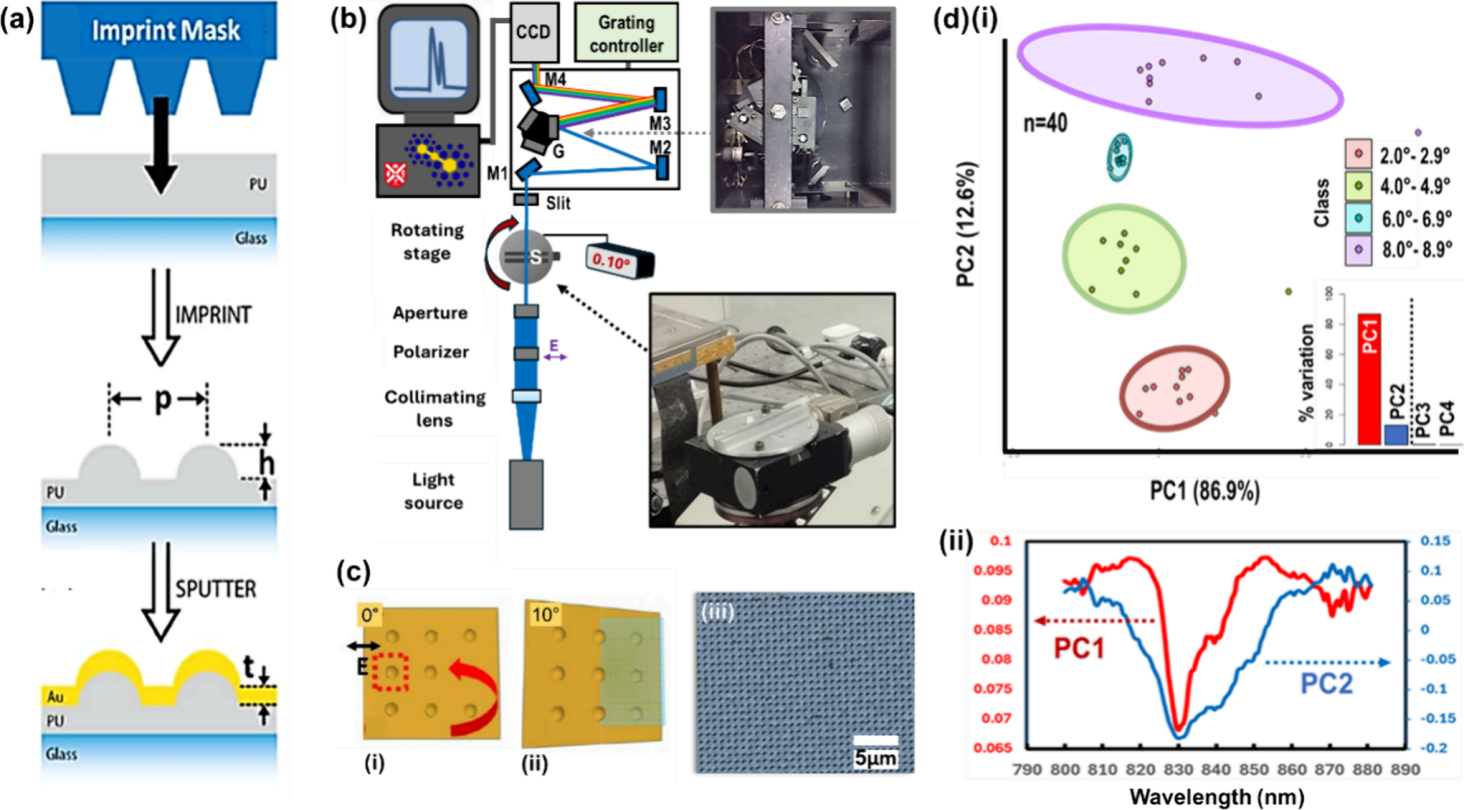
Nanodome fabrication, characterization, and principal component analysis (PCA) of white light transmission data set. (a) Nanoimprint lithography fabrication process. (b) White light transmission experimental setup. “S” marks sample position. (c) Cartoon showing rotation of nanodome structures with polarization axis marked in (i). Rotation measurements were taken up to 10° as in (ii). (iii) Optical image of iron–metallized nanodomes (also see the SI. (d)(i) PCA score plot of first two principal components (PCs) for four classes of angle ranges. Inset: PCA scree plot showing variation explained for first four PCs. The data set is white light spectroscopy in transmission mode covering the 800–880 nm spectral range. (ii) PCA loading plot for PC1 and PC2 showing wavelengths contributing most to each PC. Part (a) and (c)(i,ii) reproduced or adapted with permission from ref (63). Copyright 2019 Hardy, M., Queen’s University Belfast.

Transmittance measurements were performed in a custom-built white light transmission spectroscopy system assembled for the Smart Nano NI project (see Figure 2b) with a broadband, low-divergence laser-driven light source (Energetiq Technology Inc., USA). It was designed specifically for:

1. accurate elucidation of plasmonic modes (with optics to minimize high-angle incident photons)
2. acquisition of spectra as a function of angle (rotating sample stage)

**Figure 3:**
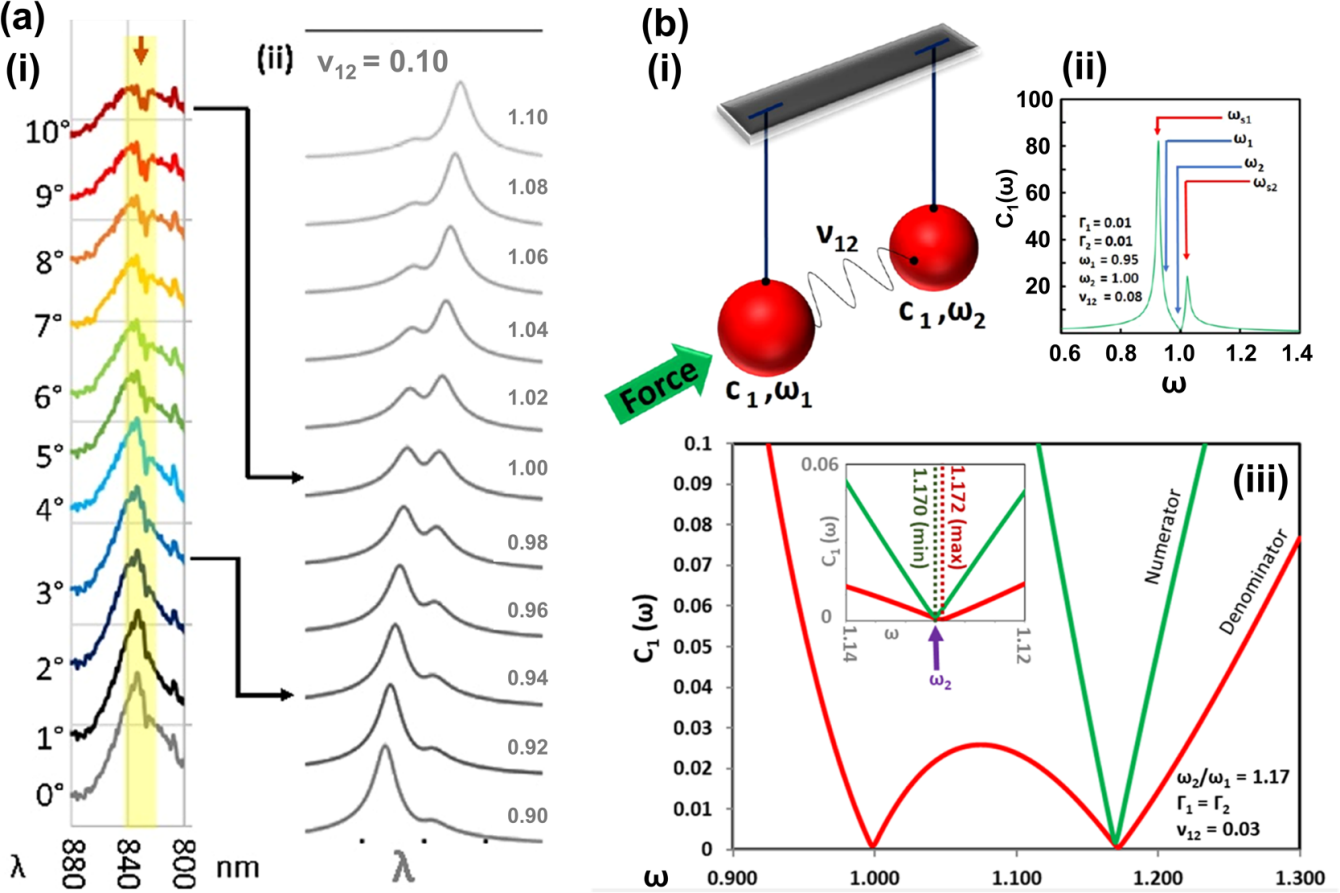
White light transmission spectroscopy Fano resonance analysis on iron nanodomes. (a) White light transmission spectra at different stage rotation angles from 0 to 10° for 800–880 nm—yellow band in (i) highlights the Fano feature—(ii) correlated with spectral patterns from a coupled two-oscillator model, as in (b)(i). Numbers on the right-hand side of (a)(ii) are respective fractional oscillator spectral positions (ω_2_/ω_1_). (b)(ii) Example spectra for the intensity of oscillator #1, *C*_1_, two oscillators at frequencies of ω_1_ and ω_2_, here set at 0.95 and 1.00, respectively, with equal line widths (Γ_1/2_) and a variable coupling parameter, ν_12_ (see eq S1). (iii) *C*_1_ intensity showing the behavior of numerator and denominator terms in the Fano equation (eq S1), with ω_2_/ω_1_ set at 1.17, Γ_1_= Γ_2_, and *v*_12_ set to 0.03. Part (b) reproduced or adapted with permission from ref (63). Copyright 2019 Hardy, M., Queen’s University Belfast.

Large-angle (2–10°) measurements and small-angle (<1°) measurements were divided by a 0° reference spectrum. The dark background was subtracted in all cases. The 0° angle position was determined by symmetry—this was determined to be at −1° via visual inspection of plotted spectra (Figure 3a), i.e., white light spectrum labeled “0°” is actually at 1° relative to impinging light beam.

### 2.5 Data Analysis

Principal component analysis (PCA) is a dimensionality reduction technique that reconstitutes variables by sequentially redefining axes in multidimensional space to explain most variance in the data set. In other words, a data set with variation “spread out” across the original variables may be transformed (or condensed) into one with new artifactual variables, which explain most of the data set’s variance in a few of these new variables (principal components). The technique thus is useful for visualizing groups/classes without any training data, i.e., unsupervised. In a spectroscopy context, the variables are the channels/wavelengths in the spectrum. The foremost principal components (PCs) thus explain most of the variation in the spectral data set. Prior to PCA, data was scaled with a standard normal variate (SNV) scale, i.e., minus the spectra data set arithmetic mean value and division by the standard deviation. PCA was then performed in RStudio version 2023.03.0 using the base R *prcomp* function.^38^ Data manipulation was performed via the R *tidyverse* package^39^ and *ggplot2.*(40) All confidence ellipses are of the frequentist definition (see Section S6). Data plotting was otherwise performed via MS Excel. A coupled oscillator model was written in MS Excel, with reference to ref (41) (Section S4 “Classical Analogy of Fano Resonances”, eq S1).

For postprocessing of Raman spectra, Renishaw WiRE 5.4 software was used.

### 2.6 Computational Modeling of Nanodomes

Finite element modeling (FEM) was conducted based on measured nanodome dimensions, with COMSOL Multiphysics (version 6.3). This was done in 2D using the Wave Optics Module to conduct a wavelength domain simulation, using either built-in optical constants for air or from Johnson and Christy.^42,43^ For the polymer substrate, polyvinyl chloride (PVC) was selected as it possessed the most similar optical properties to the substrate used in this study. Thermoforming polyurethanes and PVC have similar refractive indexes ca. 1.6 at the wavelengths of interest (633–785 nm). Scattered field with a plane wave was approximated via
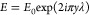
1where *E*_0_ is the initial background E-field, *i* is root −1, π is pi, *y* is the spatial co-ordinate along the axis of the plane wave propagation, and λ is the excitation wavelength. A parameter sweep was performed (200–1000 nm separation, dome edge-to-edge distance) in conjunction with a materials sweep (iron, gold, silver). The simulated surface-enhanced Raman spectroscopy enhancement factor (SERS EF) was calculated by taking the electric field to the fourth power (E^4^), as common in SERS studies.^44^ The electric field value used was the maximum value from each simulation on the basal plane, i.e., gold–air interface. For further model details, see Section S.7.

### 2.7 Raman Spectroscopy

Gelatin(e) (C_6_H_12_O_6_) from porcine skin and the heteropolysaccharide gellan gum (gel) were acquired from Sigma-Aldrich (USA) and used without further purification. Ultrahigh-quality water (18 MΩcm) type 1 was used for material formulation. Gelatin (10% w/v) was prepared by weighing out the solid portion and adding deionized water and then magnetically stirring overnight at 80 °C. Gellan gum (0.1% w/v) was prepared in the same way. To prepare the SERS substrates, solutions were drop-cast via a micropipette onto the SERS active surface to then be transferred immediately for Raman measurements.

A Raman microscope (Qontor, Renishaw plc, UK) equipped with 633 and 785 nm lasers, and 1200 lines/mm diffraction grating, was used to acquire the Raman spectra. Measurements on bare iron were acquired using 633 nm excitation; Raman measurements on iron–gold were acquired at both 633 and 785 nm excitations. The 50× objective with the pinhole in was selected for these measurements. Raman maps were acquired with a 5 μm spacing between each point. For each point, 10% laser power was selected, with 1 s exposure time over three accumulations.

### 2.8 Scanning Thermal Microscopy

Scanning thermal microscopy (SThM) is a variation of atomic force microscopy (AFM), where a small temperature probe, a nanoscale-sized thermocouple, is integrated into the AFM cantilever or tip. The SThM tip, when in contact with the sample surface, produces Joule heating by passing a small current through the probe to sense tip resistance. Depending on the thermal transport properties (thermal conductivity coefficient, *k*) of the sample, the tip resistance varies, thus producing an image depicting varying temperatures as a function of probe position.^45^ SThM offers a thermal resolution of 10 mK and a spatial resolution of 10 nm, for precise nanoscale thermal measurements.^46^

SThM images were acquired at 0.5 Hz with 256 pixels/line and 256 lines, thus providing 3.9 ms/pixel temporal resolution for the image.

### 2.9 Safety

The work herein contained no unexpected, new, and/or significant hazards or risks.

## 3 Results and Discussion

### 3.1 Large Angle Rotations (2–10°) Results

PCA on the pooled larger angle classes, with spectra truncated to a region of interest (800–880 nm), returned statistically significant differences: spatially separated 95% frequentist confidence ellipses (Figure 2d(i)). The first two PCs were determined to explain >99% of the variance in the data set, PC1 explained 86.9% and PC2 12.6%. The PCA loadings, which represent the proportion of each original variable’s (wavelengths, in our case) contribution to each PC, are graphed in Figure 2d(ii), where PC1 shows peaks around 820 and 855 nm and PC2 a peak at 830 nm (note scale difference for PC1 and PC2 respective axes, i.e., the dip/peak at 830 nm for PC1 is not significant because it has a near-zero coefficient). PC2 conveys much broader spectral features than the sharper PC1 profile. It is not clear why class 6.0–6.9° was much more tightly packed than other data classes (see blue data points/ellipse in Figure 2d(i)). Higher PCs, up to PC8, plotted in Figure S3, contrariwise showed little or no class separation.

The initial PCA results are supported by the plotting of individual spectra at different rotation angles, 0–10°, in Figure 3a(i), showing a flattening of a central peak at 830 nm wavelength and broadening of peak sidebands as predicted by the PCA loadings. This correlated with the spectral profiles from a coupled two-oscillator model (Figure 3a(ii)), explained below.

### 3.2 Small Angle Rotations (0–1°) Results

**Figure 4:**
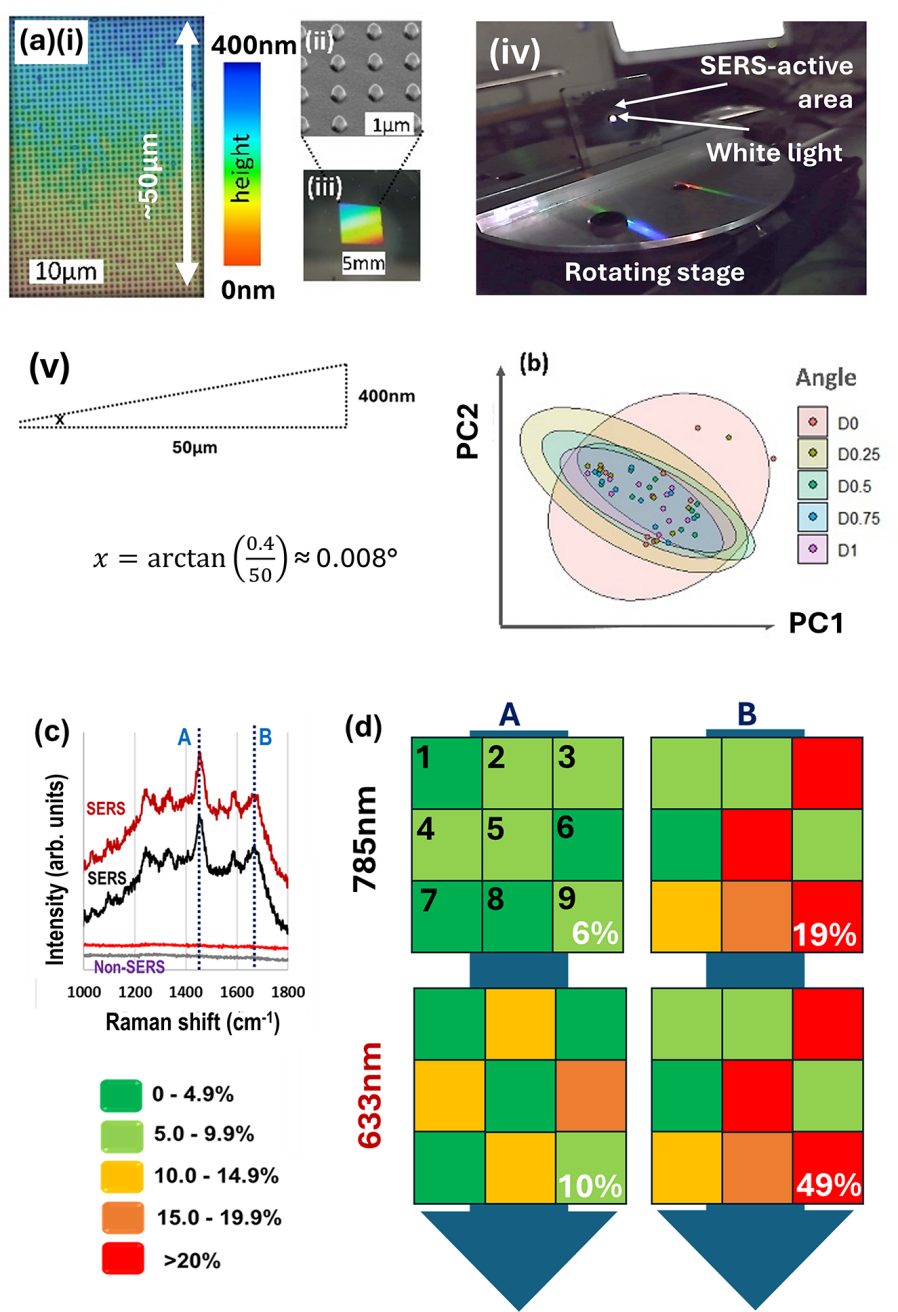
Investigation of small-angle imperfections and sensing application. (a)(i) Optical image (false color) of the nanodome surface, demonstrating the inherent flatness of the substrate—400 nm is below detectable gradient resolution of the digital microscope; (ii) zoomed-in scanning electron micrograph of nanodomes, (iii) zoomed-out optical image of nanodomes (real color/iridescence); (iv) photo of nanodomes on the rotating stage showing diffraction; (v) trigonometric calculation for the sample incline. (b) PCA score plot (PC 1 vs PC2) of spectra taken at small angles, 0, 0.25, 0.5, 0.75, and 1° rotation. *N* = 10 spectra for each angle class. Bounded areas are confidence ellipses at 95%. (c) Surface-enhanced Raman (SERS) and unenhanced Raman spectra on gold-coated nanodomes for gelatin at excitation wavelengths of 633 nm (red lines) and 785 nm (black/gray lines). Raman bands at 1473 cm^–1^ (peak “A”: gelatin CH_2_ bend) and 1666 cm^–1^ (peak “B”: amide I). (d) Background-corrected intensity maps for peaks A and B (columns), where each colored square represents one-spectrum, proximal μms-scale measurements, at wavelengths of 633 and 785 nm (rows). Color indicates percentage deviation of each measurement from the mean peak intensity key: bottom L). Percentages in white at bottom right are relative standard deviations for each set of measurements (*N* = 9).

PCA on the small angle displacement (0–1°) data showed no statistically significant separation for the five data classes, 0.00, 0.25, 0.50, 0.75, and 1.00° (95% frequentist confidence ellipses) for PC1 vs PC 2. Substrate flatness (μm–mm scale) was verified through digital optical microscopy (Figure 4a(i); see also Section S1). The analysis was performed for four different data sets:

1. The full spectrum, 400–1000 nm (Figure S3a),
2. 800–880 nm (Figure S3b),
3. 820–840 nm (Figure S3c),
4. 830–850 nm (Figure S3d).

Overlapping confidence ellipses, i.e., no statistically significant differences, were also observed for higher PCs (up to PC19 vs PC20, Figure S3) for each of the four data sets. PCA score plot data was replotted from Figure S3 and compared side by side for easy comparison in Figure S4 (same PCs, different ranges). These PC1 vs PC2 and PC3 vs PC4 score plots for the four different spectral ranges are qualitatively similar in appearance, demonstrating the lack of variation outside of the 820–850 nm range, i.e., spectral feature selections (truncations) make little difference (little meaningful information is contained outside the region around 830 nm).

### 3.3 Application Results

Raman measurements on gelatin returned clear spectra at both 633 and 785 nm laser excitation in contrast to the non-SERS case without the underlying nanostructures, which showed no spectral features (Figure 4c). Percentage relative standard deviations (100 × standard deviation/mean) for two peaks at 1473 cm^–1^ (peak “A”: gelatin CH_2_ bend) and 1666 cm^–1^ (peak “B”: Amide I) were determined to be at 6 and 19% for 785 nm laser excitation and at 10% and 49% 633 nm laser excitation, respectively (white text in Figure 4d).

### 3.4 Iron as a Biosensor Material

**Figure 5:**
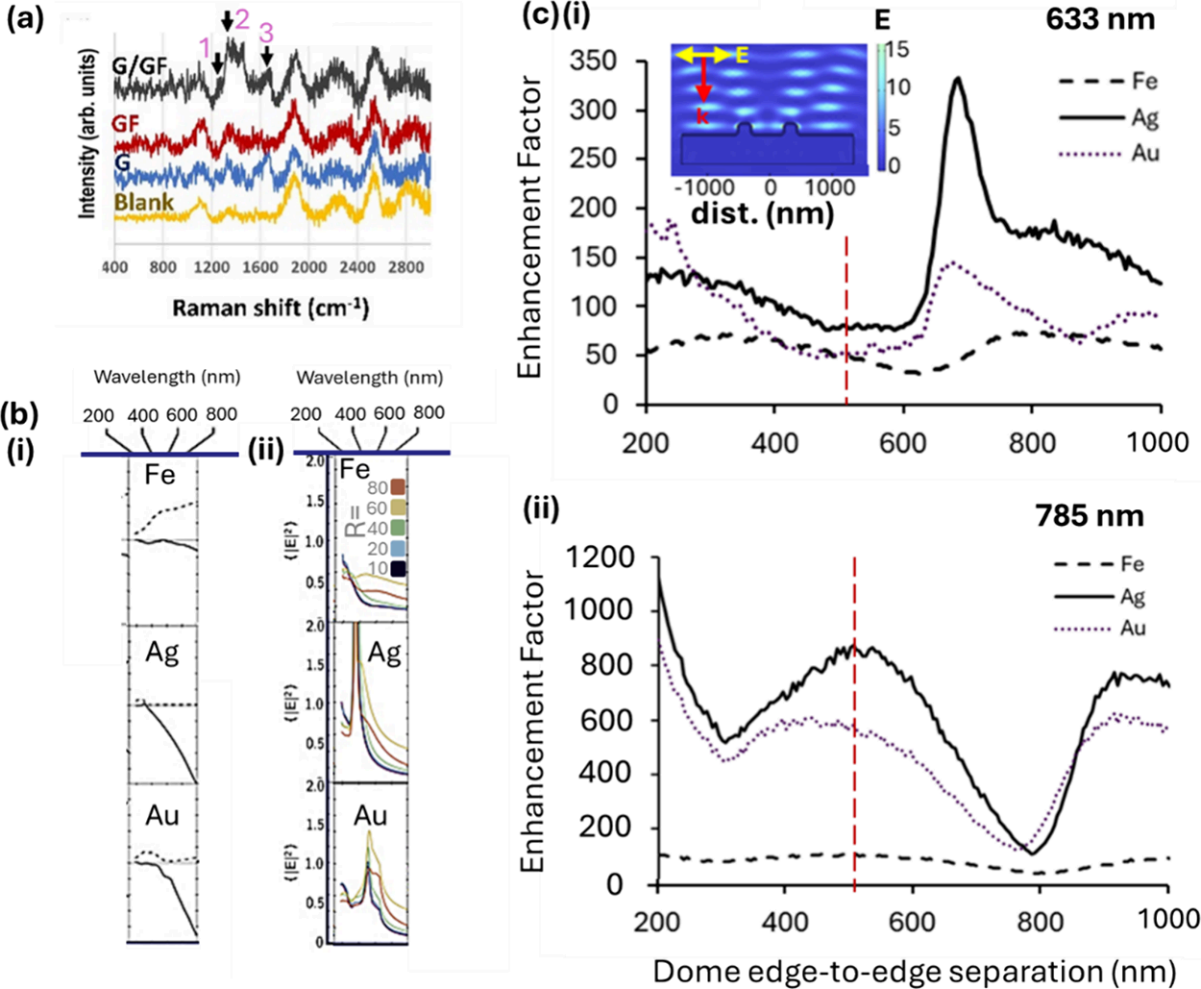
Iron biosensing: surface-enhanced Raman spectroscopy (SERS) experiment and electric field modeling (a), five-point smoothed Raman spectra (average of 100) of the blank iron-coated nanodome substrate (Blank), with gelatin (G), gellan fluid (GF), and combined (G/GF). GF bands marked (pink numbers): peak 1, amide III (1267 cm^–1^); peak 2, CH_2_ (1334 cm^–1^); peak 3, amide I (1666 cm^–1^). 633 nm laser excitation (all spectra). (b)(i) Real (solid lines) and imaginary (dashed lines) of dielectric functions for iron, silver, and gold. (ii) Local electric field intensities for spheres of radii 10, 20 40, 60, and 80 nm for iron, silver and gold. (c) Numerical electric field modeling of iron-, silver-, and gold-coated nanodomes (metal thickness 30 nm all cases). (i, ii) SERS enhancement factor (EF) for varying dome-to-dome spacings at (i) 633 nm and (ii) 785 nm excitation. All models 2D, two dome models; separation is given dome edge to edge. Simulated EF is calculated via *E*^4^ from the maximum *E*-field value observed at the gold–air interface. Red vertical dashed lines indicate dome edge-to-edge spacing used in this study (510 nm, i.e., 790 nm dome center to center). Note, not including the 30 nm gold layer—see Section S7 for details. Inset in (c)(i) shows the representative COMSOL plot for Fe with 633 nm excitation (for full simulations, see Figure S8). All material data from Johnson and Christy (Ag and Au) as well as Johnson and Christy (Fe), refs (42, 43). Part (b) adapted from ref (54). Gutiérrez et al., Plasmonics beyond Noble Metals: Exploiting Phase and Compositional Changes for Manipulating Plasmonic Performance. J. Appl Phys 2020, 128 (8), 080901. 10.1063/5.0020752 with the permission of AIP Publishing.

**Figure 6:**
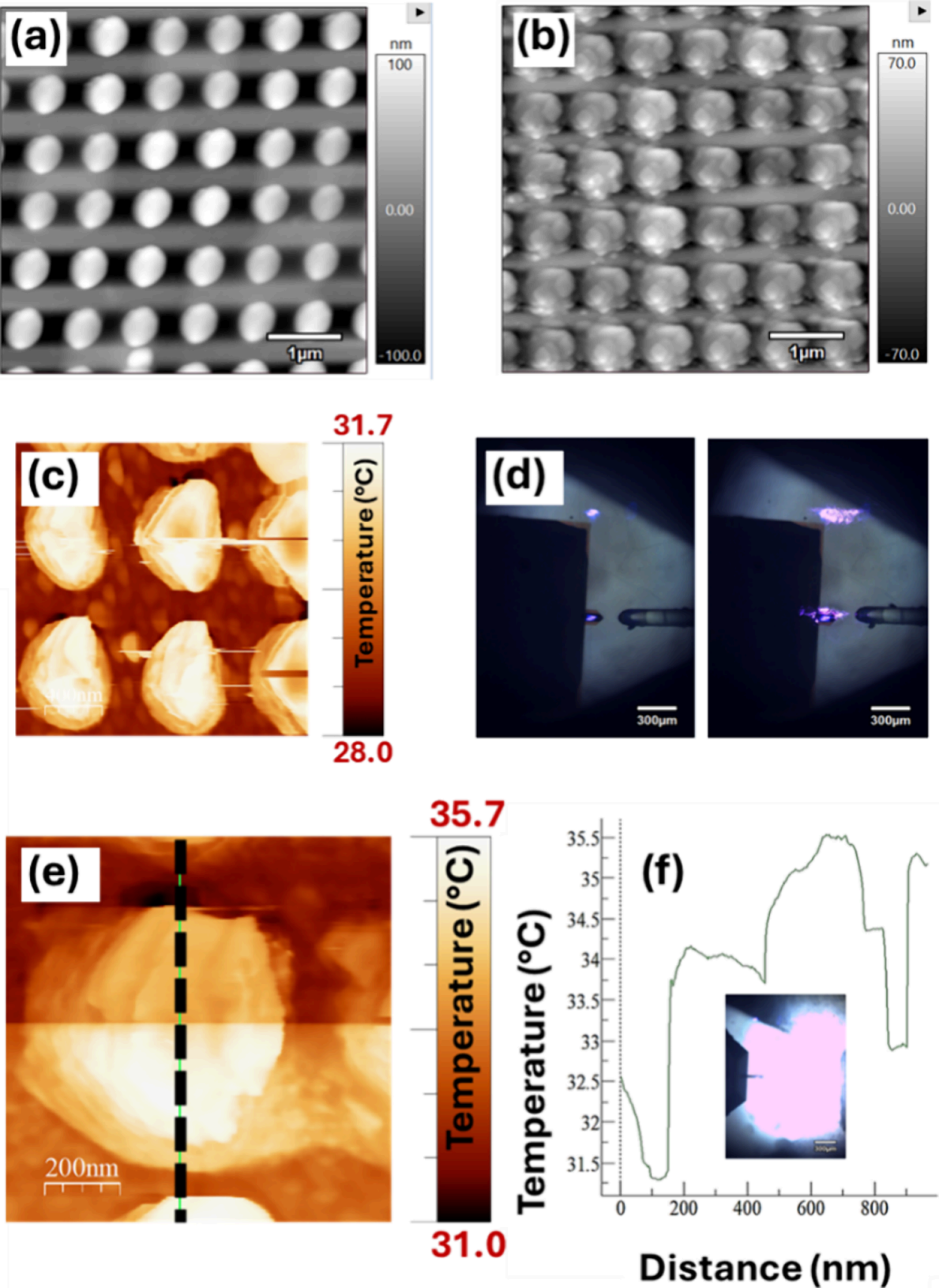
Nanothermal response via scanning thermal probe microscopy. (a, b) Atomic force microscopy images of Fe nanodomes in (a) tapping and (b) contact operation modes. (c) Scanning thermal probe microscopy (SThM) image (area ∼3 μm^2^). Top illumination temperature plot with (d) a fiber optic light source (L: off; R:on). (e) SThM bottom illumination temperature plot of single nanodome with light intensity increased from 0 to 100% and (f) graphed along dashes in (e). Inset: bottom illumination with broadband white light.

In the final part of the experiment, iron was explored as an alternative biosensing material. Perhaps surprisingly, clear Raman peaks associated with gelatin and gellan fluid were visible when applied to the bare iron surface (Figure 5a). Modest local temperature increases around the nanostructures were observed with a scanning thermal probe microscopy setup—up to 5 °C (Figure 6) (∼1 μm^2^). Individual nanodomes appeared to remain intact at this level of local heating. This was ∼2× that of the same nanostructures coated in gold (2–3 °C) over a similar area ∼1 μm^2^ (see Figure S6). The metal was verified as iron via electron X-ray diffraction spectroscopy (Figure S5).

### 3.5 Large-Angle Data Set Discussion

As a starting point, we note that the peaks in the large-angle data set (Figure 3a(i)) can be understood as resulting from the repeating structures. The feature at 830 nm corresponds to a Bloch mode of the system, which are excitations associated with periodic arrays and apply to systems in general not only nanoplasmonic—here, specifically, a propagating surface plasmon polariton (SPP) mode, a moving hybrid photon–electron excitation. The spectral position of Bloch excitations is given by the grating equation:
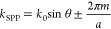
2where *k*_SPP_ is the SPP wavevector, *k*_0_ is the free-space light wavevector (=2π/λ_0_), θ is the incident angle of the light, *m* is the order of diffraction, and *a* is the grating period.^17^ The period, when it matches the wavelength, is an effective tool to phase match and thus efficiently couple the impinging light photons into the SPP mode. Where the light is normal to the surface,
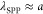
3

Although we discuss the Bloch mode wavelength corresponding to the SPP wavelength, this is an approximation: The SPP resonance wavelength also depends on material (Au@800 nm ϵ_1_ ∼ −24; ϵ_2_ ∼ 1.5; Fe@800 nm ϵ_1_ ∼ 2.7; ϵ_2_ ∼ 20^42^). The larger real part (ϵ_1_) of the dielectric function (dielectric constant) for iron, in comparison to gold, means that SPPs in iron form at longer wavelengths (smaller wavevector, *k*_SPP_). Similarly, and underdiscussed, is the effect of the applied analyte medium on the SPP resonance position, here, gelatin, with refractive index properties comparable to water, although normally in SERS studies, the analyte applied is in low volume and low concentration, and resulting plasmonic shifts can be largely neglected.

### 3.6 Interpreting PCA Loadings, in Relation to Physical Phenomena

PCA loading plots (Figure 2d(ii) convey the variables in the original data set, which contribute most to the reconstituted variables: the PCs. The axis of maximal variance, PC1, does not permit class separation; on the other hand, PC2, despite representing much less variation in the data set, does. Here, there are also broadening side bands (+ part of the secondary vertical axis, PC2) and complementarily, the damping of the central peak (− part of axis, PC2). In interpreting PCA loading plots, the sign is arbitrary, but the magnitude represents the extent to which an original variable (wavelengths, here) contributes (thus, note zero position on the PC2 axis in Figure 2d(ii)).

The PCA loading findings are thus instructive and are explored further by plotting of the original white light transmission spectra in Figure 3a(i), where a reduction in central peak intensity (840–845 nm) above the baseline is accompanied by a subtle dip appearance ∼850 nm as the angle of rotation is increased at 0–10°. In alignment with the PCA loading prediction, there is a concomitant broadening of the peak sides as the spectral feature “flattens out” with increasing angle. This feature can be understood as a Fano resonance, a well-understood phenomenon where two system resonances interact, in its purest form, one that is comparatively narrow and one that is broader, to form an asymmetric line shape.^47,48^ The system can be understood by a simple, classical physics, two-coupled oscillator setup,^41^ with a force applied to one of the oscillators, where the respective oscillator intensities, *c*_1_, *c*_2_, natural frequencies, ω_1_, ω_2_, and line widths, Γ_1_, Γ_2_, can be modeled, with a coupling constant, ν_1.2_ (Figure 3b) (see eq S1). A full discussion on Fano resonances in nanoplasmonic systems is given in ref (47).

Here, the presence of the Fano feature is likely as a result of the interaction between the propagating SPP excitation associated with the periodic array of nanostructures and a localized surface plasmon polariton (LSP) excitation of individual nanostructures, the position of which (for both modes) spectrally shifts as the angle of the incident photons is changed. Thus, we postulate that increasing the angle of the light substrate causes an overlap (interaction) of the two system modes, not evident at 0°, where we see only the SPP mode. In a practical sense, this kind of Fano phenomenon is likely not to be beneficial here, where a plasmonic sensing platform may no longer be optimized for the desired laser wavelength—the energy in the near field is being redistributed to the detriment of the plasmonic sensor. Regardless, such large angular discrepancies between the incident laser light and substrate plane are unlikely to be present in reality. The real benefit to this kind of model is in understanding and prediction of spectral resonance positions by visual correlation of spectral features to the output from the coupled two-oscillator model (Figure 3a(ii)). This, then, potentially allows estimation of the spectral position of the dome LSP modes based on the spectral position of the SPP modes (see eq S1) and would make interesting further study.

### 3.7 Meaningful Variance in PCA

The study herein also brings forth some broadly applicable points about using PCA in the understanding of spectra: first, a distinction between maximal variance, which the foremost PCs describe by definition, and meaningful variance, which is frequently confounded with the former. Often, the greatest varying spectral features offer the most discriminatory power between classes, which is to be expected, because classes have been prescribed prior to experiment, which have some sort of difference, e.g., pathological vs healthy tissue samples. However, this is not necessarily true and differences within classes, intraclass variance, can dominate. This is the case in the white light transmission large-angle study here, where PC1, although representing most of the difference in the data set, does not lead to statistically significant class separation. This is apparent in the projection of the data points in the PC1 vs PC2 PCA scores plot (Figure 2d(i)) onto the PC1 (horizontal) axis. Instead, the true class discrimination, the interclass variation, is contained within PC2, where the separation between classes is maintained if the data is projected onto the vertical PC2 axis.

This critical point would appear to be often overlooked in the literature, despite the ubiquitousness of PCA as an initial pattern-finding unsupervised data analysis technique, in spectroscopy studies and beyond. Instead, focus frequently falls (merely) on how many PCs to retain, which are then fed into a supervised classification algorithm and presented as a hyphenated model, e.g., PCA-LDA and PCA-SVM, which make use of (linear) discriminant analysis and support vector machine classifiers, respectively. Clustering in the form of a *K-*nearest neighbor approach can be similarly used PCA-KNN.^49^ PC retention criteria include, for example, a visual scree test, i.e., looking for inflection points in a scree plot of PC variance (inset of Figure 2d(i)). The eigenvalue-greater-than-one criterion is also employed; specifying any retained PCs must explain more variance than, on average, explained in the original data set. Others simply look at cumulative variance as a way to decide how many PCs to retain, normally set at 95% cumulative variation explained. None of these methods, however, consider meaningful variance.

Second, and similarly, the degree of variance explained must be considered, alongside some knowledge, if possible, of what is changing. For instance, albeit not statistically significant, a pattern of progression for the small-angle classes is apparent in PC9 vs PC10 (Figure S2a). However, PCs beyond PC5 explain a miniscule part of what is varying in the data set (<0.1%) and thus should be disregarded as spectral noise. This is in fact a use of PCA, denoising by removal of higher PCs, identified in the original PCA paper by Wold et al.^50^ Moreover, the PCA loadings, which convey the original variables contributing most to each PC, need to make sense. In a spectroscopy context, white light peaks should be seen to correspond to bone fide plasmonic resonances. The loadings for PC9 vs PC10, for instance, display erratic features not attributable to any physical phenomena. In a Raman spectroscopy context, loadings features should correspond to known Raman transitions.

### 3.8 Iron and Other Alternative SERS Media

The original intention of this study was to use the ferromagnetic properties of iron, as an underlayer, to tune the resonance position of the plasmonic resonances on the gold top layer, i.e., magnetoplasmonics, via spin–orbit coupling (and Lorenz force drift),^51^ which has promise for biosensing applications.^52^ However, we noted the enhancing properties of the (bare) iron surface, which permitted the identification of some gelatin/gellan fluid peaks (Figure 5a), which, while modest, is useful. This leads to a question of what magnitude of enhancements are truly necessary in SERS studies. While ferromagnetic materials have been used elsewhere as plasmonic enhancers, they have been largely deemed insufficient on their own, instead requiring a gold or silver overcoat. Although gigantic enhancements, say up to 10^8^, are routinely reported, this increase in the Raman signal is unlikely to be necessary in most scenarios where concentrations are still relatively high, i.e., far from the single/few-molecule detection regime. It may thus be an advantage to employ alternative plasmonic enhancers, such as iron, which can confer other benefits, such as plasmon resonance tunability (via magnetic field application). Moreover, we note that our initial numerical modeling suggests that for the dome geometry and spacing used here, iron can offer enhancements comparable to gold and within a factor of 2 to silver at 633 nm excitation, and within an order of magnitude to gold and silver at 785 nm (see dashed red lines, Figure 5c), albeit more detailed models will be needed.

In some cases, the uses of some different plasmonic metals might fall naturally within the experimental remit, e.g., lithium in battery studies,^53^ but which is modest, for example an enhancement factor (EF) of 30× (at 638 nm excitation) in the battery studies of Tang et al. or lower still, e.g., due to the highly reductive nature of lithium, forming an oxide layer.^23^ Ni and Co are the other common ferromagnetic materials, which can support plasmons in the visible, with more negative real dielectric functions than iron, but larger imaginary parts, i.e., more lossy.^54^ Away from ferromagnetism and tunability, Al is a well-known option for UV excitation of plasmon polaritons.^55^ A recent review proposes Mg as useful in nanomedicine, being short-lasting rather than accumulating in the body, and alongside Al and Cu, which are cost effective.^56^ Moreover, there is a recent focus on dielectric SERS,^57^ which evokes chemical enhancement effects and confers benefits in terms of cost and ease of production, for instance in vanadium oxide^58^ and titanium nitride platforms (EF = 170).^59^ All-dielectric nonmetallic SERS surfaces, which offer potential for 3D SERS, could be “invaluable”.^60^ Moreover, the iron substrate analyzed in our study gave a flat background signal in the region of interest Figure 5a). Contrariwise, the gold nanodome-coated sample gave a much more complex background signal, which is more cumbersome to subtract, leading to, in our study, larger than expected relative standard deviations for the prominent amide I peak at 1666 cm^–1^ (peak B in Figure 5) (49% RSD at 633 nm; 19% RSD at 785 nm), which is positioned at the place of a precipitous background signal drop-off. On the other hand, an analysis of the amide I 1666 cm^–1^ peak for the bare iron substrate, based on the selection of 10 random points across the mapped area, returned an RSD of 13% (without background subtraction).

Beyond benefits in magnetic-induced resonance position tuning, and issues relating to material costs, the scanning thermal probe microscopy study in Figure 6 shows that alternative materials can also have more suitable thermal properties. Iron has a well-known large specific heat capacity with respect to gold (∼0.45 vs 0.13 J/g·K), meaning that the iron might be expected to heat up more slowly than gold. However, this was not observed. The iron domes herein returned a relatively large temperature increase over the area of one nanostructure (4–5 °C); a gold comparison measurement over a similar sample area, i.e., 1 μm^2^ (Figure S6), had a temperature increase of around 2 °C. This perhaps indicates that despite a lower specific heat capacity, iron still heats up more readily than gold under certain circumstances, i.e., white-light illuminated, a property probably related to its lower reflectivity and lower thermal conductivity than gold. We note the increased thickness of the gold comparator sample vs the iron thickness (60 vs 30 nm). In preliminary study, we have noted deformations of nanodome structures (Figure S7c), presumed to be a result of laser damage. This was surveyed via scanning electron microscopy, but strategies to monitor this by more convenient means, or to remove the possibility of laser damage altogether, would be useful.

### 3.9 Application and Small-Angle Study Discussion

The detection of gelatin and gellan fluid pertains to their use as an approximation to the mucosal lining, and a new compound in coronavirus nasal sprays, respectively, as we have recently documented in ref (61). Their interaction is an ongoing experiment, i.e., does the preventative constituent, gellan fluid here, change in any way as it adheres to the nasal lining surface? Measurements of the %RSDs for the 1473 cm^–1^ gelatin CH_2_ bend (6 and 10%) are suitable for analytical purposes.^62^ This is, in fact, the real selling point of ordered arrays, where a more uniform SERS signal is possible spot-to-spot and between batches. Ordered SERS substrates are of a different character to SERS media, which are less reproducible but afford gigantic enhancements.^28^ Previously, we have examined a range of EFs in similar structures, observing enhancements of up to 10^6^ times versus a non-SERS case.^63^

In principle, very-small-angle mismatches are detectable via white light transmission coupled with data analysis strategies; the limitation in our study here is the system setup. More PCs could be included to achieve separable classes but at the risk of overfitting models and concomitantly reducing model robustness (i.e., future accuracy, predictability, by including noise). However, it may not be beneficial to detect small singular mismatches; instead, tolerances for acceptable discrepancies should be determined. A similar limitation is in determining the 0° angle (as detailed in Section 2) where there is an uncertainty of 1–2°, which is problematic for determinations in the small-angle regime but could be remedied with improvements to the system setup.

### 3.10 Further Discussion and Experiments

Fano resonances have proved useful for sensing purposes.^47,64^ Further analysis of the system would be useful, perhaps supported by angle-varied numerical models. Iron can be used to aggregate nanoparticles in SERS for hotspot generation^65,66^ when an external magnetic field is applied—it may be interesting to combine a tunable ferromagnetic SERS substrate with moveable ferromagnetic nanoparticles for the same purpose; to our knowledge, this experiment has not been done.

Here, the angle-resolved measurements were in white light transmission, but angle-resolved Raman spectroscopy measurements are also interesting. We note that off-angle SERS may be underappreciated in general, but appears in the old literature^67^ and extends to microscope objective choice, where high numerical aperture (NA) lenses have larger solid (cone) angles and thus excite (different) plasmonic modes with more obliquely incident photons. Normally, the conversation around objective lens selection would seem to be confined largely to spot size, where higher-NA, higher-magnification lenses have smaller focused spot sizes on the sample surface, but this is only truly relevant for situations where there is sample inhomogeneity. In addition, it can be noted that NA can also be important where the SERS analyte scatters anisotropically, thus affecting the magnitude of the collected Raman scattered light.^68^

This study lays the foundation for a method for rapid and accurate determination of defects in nanoimprint-produced plasmonic substrates—if the metal coverage is not so thick to be opaque to an incident light source. Other discrepancies exist, for example missing nanostructures, or more commonly, differences in structure height due to incomplete depression of the submaster template into the polymer. This latter defect is especially problematic in the low-production research environment, where small numbers of samples are made without automation. In our own efforts, we have noted structure heights closer to 180 nm with a 200 nm deep stamp. Via scanning electron microscopy, roughness around some nanodome bases has been observed (see Figure S7a), which could have a significant effect of the SERS signal, rendering the SERS substrate more “hotspot-dominated”,^29,69,70^ i.e., with a larger enhanced Raman signal but lower reproducibility, and thus less suitable for analytical applications. Roughness defects are unlikely to have significant signature in the far-field spectra, and thus unlikely to be detectable via white light spectroscopy,^29^ although they may be discernible via ellipsometry in some cases.^71^ We note that this defect also permits more analyte molecules to adsorb to the surface, as postulated in the original SERS experiments of pyridine at electrochemically roughened silver electrodes, but the enhancement from rough surfaces due to areal increase is probably 10–100× at most.^60^ Similarly, SEM has also shown some sample “streaking” and “warping” (Figure S7b–d) where we think that the use of high-power laser at 785 nm, in order to get a sufficient signal due to the 1/λ^4^ dependence of the Raman signal with wavelength, coupled with the low thermal conductivity of polyurethane, has caused sample damage. We think many of these defects should be detectable with the white light and data analysis strategy.

Efforts are ongoing for improvements to the NIL fabrication process, for instance, in mask-to-wafer alignment and force applied.^72^ In addition, NIL has applications beyond biosensing, but to make inroads in application spaces such as CMOS devices, improvements to not only production processes but also subsequent quality assurance testing must be sought.^73^ Here, we have proposed optimized white light transmission with unsupervised machine learning as a means for rapid assessment of substrate fidelity. The crucial aspect of the experiment is the transmission arrangement, rather than the more conventional setup in reflection, where transmission affords sharper peak profiles.

## 4 Conclusions

We have presented a method for the rapid identification of imperfections in nanoimprinted substrates demonstrating differences in the large angle regime, which could be extended to smaller angle displacements with system adjustments. The sensor showed a reproducibility as low as 6%, which is likely to be lower with improvements to the background subtraction method. The study also draws important side conclusions. First is on the nature of PCA data interpretation: (1) That maximum variance is not necessarily the most meaningful and (2) the PCA-transformed data, specifically the PCA loadings, which show the most varying variables, should be mapped to changes that make physical sense (where possible). Second is on the importance of considering alternative SERS materials, which may provide small yet useful enhancements. Nanoscale thermal effects in plasmonic sensing were highlighted—here, all nanostructures appeared to remain intact, even on the iron-coated surface, despite an unexpectedly large temperature increase with respect to the gold-coated nanodomes.

Herein, we have focused on one specific kind of nanoimprint imperfection: substrate plane-light mismatch, but other potential imperfections exist, for example, missing nanostructures, roughness, or laser-damaged samples, where the laser power has been set too high. Even when these unwanted features are detected, it is unclear how global they are across the sensor area (on any one chip) and across sensor chip batches. The rapid white light and data analysis techniques presented here could help answer that. Various defects all will have different spectral profiles, which may be detectable by machine learning strategies.

## Data Availability

Available on request.

## Supporting Information Available

Fabrication (metallization), dimension sizes, Fano theory, data analysis (full principal component analysis plots), scanning thermal probe microscopy reference measurement, other possible defects (scanning electron micrographs), and numerical modeling of nanodome structures (PDF)

## Notes

We declare that this research did not require Ethics approval.

## Notes

All the authors of this manuscript consent to its publication.

## Notes

AI was not used in the production the figures or text in this manuscript.

## Notes

https://creativecommons.org/licenses/by/4.0/deed.en

## Notes

No special safety concerns
