# Supplementary material for: White Light Transmission Spectroscopy for Rapid Quality Control Imperfection Identification in Nanoimprinted Surface-Enhanced Raman Spectroscopy Substrates
Source: ACS Meas Sci Au. 2025 Mar 1;5(2):250–63. doi: 10.1021/acsmeasuresciau.5c00003 (PMC12006957; doi:10.1021/acsmeasuresciau.5c00003)
Supplement: Supplementary file 1 — tg5c00003_si_001.pdf [file tg5c00003_si_001.pdf]

–Supporting Information–

White light Transmission Spectroscopy for Rapid Quality Control Imperfection Identification in Nanoimprinted Surface Enhanced Raman Spectroscopy Substrates

Mike Hardy[a,*], Hin On Martin Chu[b,c], Serene Pauly[a,d], Katie F. Cavanagh[a,e], Breandán J.F. Hill[a,f], Jason Wiggins[a], Alina Schilling[a], Pola Goldberg Oppenheimer[b,c], Liam M. Grover[c], Richard J. Winfield[g], Jade N. Scott[a], Matthew D. Doherty[h], Ryan McCarron[h], William R. Hendren[a], Paul Dawson[h], Robert M. Bowman[a].

[a]*Smart Nano NI, Centre for Quantum Materials and Technologies, School of Mathematics and Physics, Queen's University Belfast, BT7 1NN, UK*
[b]*Advanced Nano-Materials Structures and Applications Laboratories, School of Chemical Engineering, University of Birmingham, B15 2TT, UK*
[c]*Healthcare Technologies Institute, Institute of Translational Medicine, Mindelsohn Way, Birmingham, B15 2TH, UK*
[d]*Photonic Integration and Advanced Data Storage CDT, James Watt School of Engineering, University of Glasgow, G12 8QQ, UK*
[e]*Yelo Ltd, 20 Meadowbank Rd, Carrickfergus, Co. Antrim, BT38 8YF, UK*
[f]*Causeway Sensors Ltd, 63 University Rd, Belfast, BT7 1NF, UK*
[g]*Tyndall Micro Nano Electronics, Tyndall National Institute, University College Cork, Lee Maltings, Cork, T12 R5CP, Ireland*
[h]*Centre for Nanostructured Media, School of Mathematics and Physics, Queen's University Belfast, BT7 1NN, UK*

*mhardy04@qub.ac.uk / smartnanoni.com

S1. **Estimation of inherent incline of nanostructure surface plane**

Approximation of sample flatness from digital microscopy (Figure4(a)). Purported height span = 400nm

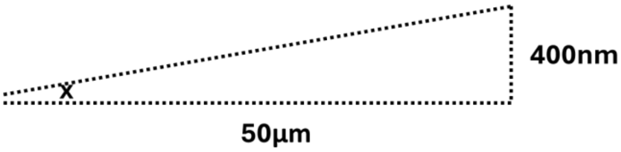

(essentially flat, below limit of detection for optical microscopy) over a measured lateral span of ~50µm):

$\tan(x)=0.4/50$

$x=\tan^{-1}(0.008)$

$=0.008°$

Measurements of dome period (from optical images as in Figure2(c)(iii)). Dome period (center to center) as fabricated specification=800nm (end point to end point ~ 520 nm). As measured via optical images (mean of 25 period span): 0.79, 0.79, 0.79 i.e. 3 measurements of 25 periods. Overall mean dome to dome distance = 0.79 µm (790nm – end point to point = 510 nm) i.e. below the 800nm prescribed. Any offset angle in planar incline will reduce the actual dome-to-dome distance (period):

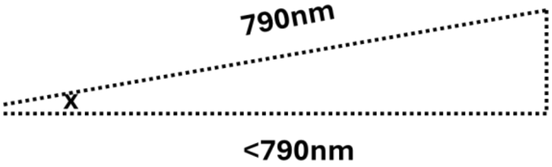

S2. **Choice of sample**

Nanodome Replicate C was chosen because the sample appeared to be most pristine. Replicates A and B showed some 'bubbling' of the polyurethane material substrate. Thermal scanning microscopy measurements were performed post-main experiments and thus Replicate C was used for this exploratory analysis of the thermal properties of the iron-coated sample.

There was some variation in nanodome height between replicates due to incomplete depression of the submaster stamp into the polyurethane. The submaster template had a height of 200 nm. Actual heights were between 180nm-200nm. Similarly, we note the relaxation of the polyurethane during the fabrication process, which, in fact, gives the domes their more-rounded ‘dome-like’ shape (the submaster template is a cube shape). There is also some variation in this process, which we plan to quantify in further study.

S3. **Compositional Analysis**

The iron-coated calibration sample was evaluated with energy dispersive x-ray spectroscopy (Figure S5), showing a high Fe yield, alongside Si and O from the glass slide. We note the presence of smaller percentages of various elements (Na, Cl, Mg), which probably pertains to handling. Interestingly, we had to optimize the x-ray current significantly in order to record a notable Fe percentage (26%), presumably because the signal is too low if the current is too low and too many x-rays pass through the thin Fe surface layer if it is too high. The film was observed to be a dull grey color, as expected (silver is expected to be lighter/brighter) and it did not scratch easily (unlike silver and gold). The later characteristic could be another benefit to using iron films.

S4. **Classical Analogy of Fano Resonances**

The theory of Fano resonances can be understood with two coupled oscillators with a force applied to one of the oscillators (Figure3(b)):

$$F(\omega) = A_F \frac{\omega_2^2 - \omega^2 + i\Gamma_2\omega}{(\omega_1^2 - \omega^2 + i\Gamma_1\omega)(\omega_2^2 - \omega^2 + i\Gamma_2\omega) - v_{12}^2} \quad \text{(S1)}$$

Where ω_1 and ω_2 are oscillator frequencies, with linewidths Γ_1 and Γ_2, and a coupling parameter, v_{12}. A_F is the Fano amplitude. *i* is the imaginary unit. See Ref [1] (Joe et al. 2006)

S5. **Metallization of iron, iron-gold and gold films**

There were three different types of thin film substrates measured in this study: 1. Iron (only), 2. gold on iron, and 3. gold (only) for the scanning thermal probe microscopy experiments. Details of the iron film thermal evaporation at Queen’s University Belfast and overcoat of the gold layer used for the subsequent SERS measurements are included in the main text (Section 2.2. Nanostructure Metallization). For the tangential experiments with Scanning Thermal Microscopy, a further set of nanostructures was used that had a different (dome-to-dome) period (this is inconsequential because the thermal response of individual nanodomes was monitored). The samples were kept under vacuum until use.

The metallization (sputtering) was performed at Queen’s University Belfast with a Lesker sputtering system with the following conditions: 60nm of Au, deposited at a rate of 1.6 Angstroms/s. Base pressure of the chamber was $8x10^{\wedge-9}$Torr and the Ar sputtering pressure was 0.8 mTorr. Samples were deposited at room temperature.

S6. Data plots – statistical interpretation

All confidence ellipses (Cis) are plotted as *frequentist* CIs. This means that statistical parameters e.g. arithmetic mean, are viewed as being fixed, albeit unknown. The CIs can thus be interpreted as follows: if

the experiments were to be repeated many times, then the chance that a parameter lies within the confidence ellipse would be 95%.

In contrast, *Bayesian* CIs are a different statistical framework, where a CI in a Bayesian context represents a 95% chance that the parameter lies within the CI.

S7. Computational Modelling of Nanodomes: Additional Information

2D domes (two-dome model) were 200nm×200nm (width and height) (Figure S8). A 30nm gold layer was added around the domes. The dome center-to-center in the actual experiments was 800 nm, or 790 nm measured, as noted above. This translates to a dome edge-to-edge of 510nm (dome sides are 200 nm, thus hypotenuse distance (‘2q’) is sqrt(200^2) ~ 282 nm, and half hypotenuse distance (‘q’) is then 141 nm. The model, with distance 200 nm (100 nm half distance) is short of the actual distance, but the point of the model is to investigate the materials’ effect (Fe, Au, Ag) alongside nanostructure separation (200nm-1000nm). Note, the separation in the models does not include the metal thickness (30 nm Au).

Please see Figure S9 for explanative schematic.

The distance around the domes in the COMSOL model was set at 12 μm high and 12.5 μm wide. The perfectly matched layer (PML) at model boundaries was set to be three times the wavelength.

S8. Additional conclusions

The study also reports on two important additional results: 1. The interpretation of PCA data can be crucial when variation exists *within* classes i.e. intraclass variance. While this phenomenon is far from uncommon, it would appear to be understated in the spectroscopy/biosensing literature, and may need to be emphasized in emerging fields where there is inherently a significant amount of unintended variation in sample composition between samples, such as in saliva, an emerging diagnostic biofluid [2]. And secondly, that alternative sensing materials may provide useful sensing properties despite reduced sensitivity. This area has recently spawned a lot of interest, but alternatives to silver and gold (in SERS) have yet to make a commercial indent. Modest enhancements in SERS perhaps deserve more attention, especially when the materials used confer significant benefits over the traditional coinage metals. This has also been highlighted in a SPR-SERS combined study, for instance, where low enhancements of 10^2 are given as the SERS increase at the planar metal interface (in a Kretschman SPR configuration [3].

References

[1] Joe, Y. S.; Satanin, A. M.; Kim, C. S. Classical Analogy of Fano Resonances. *Phys. Scr.* **2006**, *74* (2), 259–266. https://doi.org/10.1088/0031-8949/74/2/020.

[2] Hardy, M.; Kelleher, L.; de Carvalho Gomes, P.; Buchan, E.; Chu, H. O. M.; Goldberg Oppenheimer, P. Methods in Raman Spectroscopy for Saliva Studies – A Review. *Appl. Spectrosc. Re.v* **2022**, 57 (3), 177–233. https://doi.org/10.1080/05704928.2021.1969944.

[3] Meyer, S. A.; Le Ru, E. C.; Etchegoin, P. G. Combining Surface Plasmon Resonance (SPR) Spectroscopy with Surface-Enhanced Raman Scattering (SERS). *Anal. Chem.* **2011**, 83 (6), 2337–2344. https://doi.org/10.1021/ac103273r.

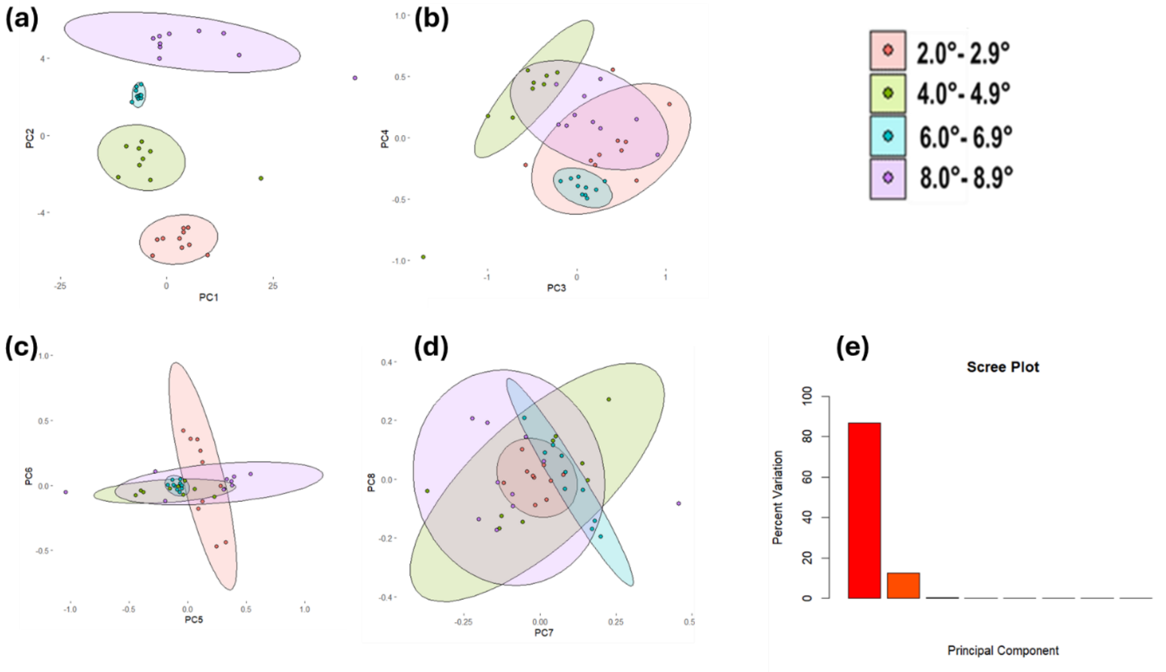

Figure S1. Principal component analysis (PCA) scores plots for principal components (PCs) for four classes of angle ranges. Dataset is white light spectroscopy in transmission of iron nanodome sample (800nm-880nm spectral range) (a) PC1 vs. PC2 (b) PC3 vs. PC4 (c) PC5 vs. PC6 (d) PC7 vs PC8. (e) Scree plot showing percentage variation for first eight PCs (as presented in parts (a)-(d). N=10 for each class (angle range). Confidence ellipses are set at 95% confidence. Expanded dataset from Figure 2(d) (main text).

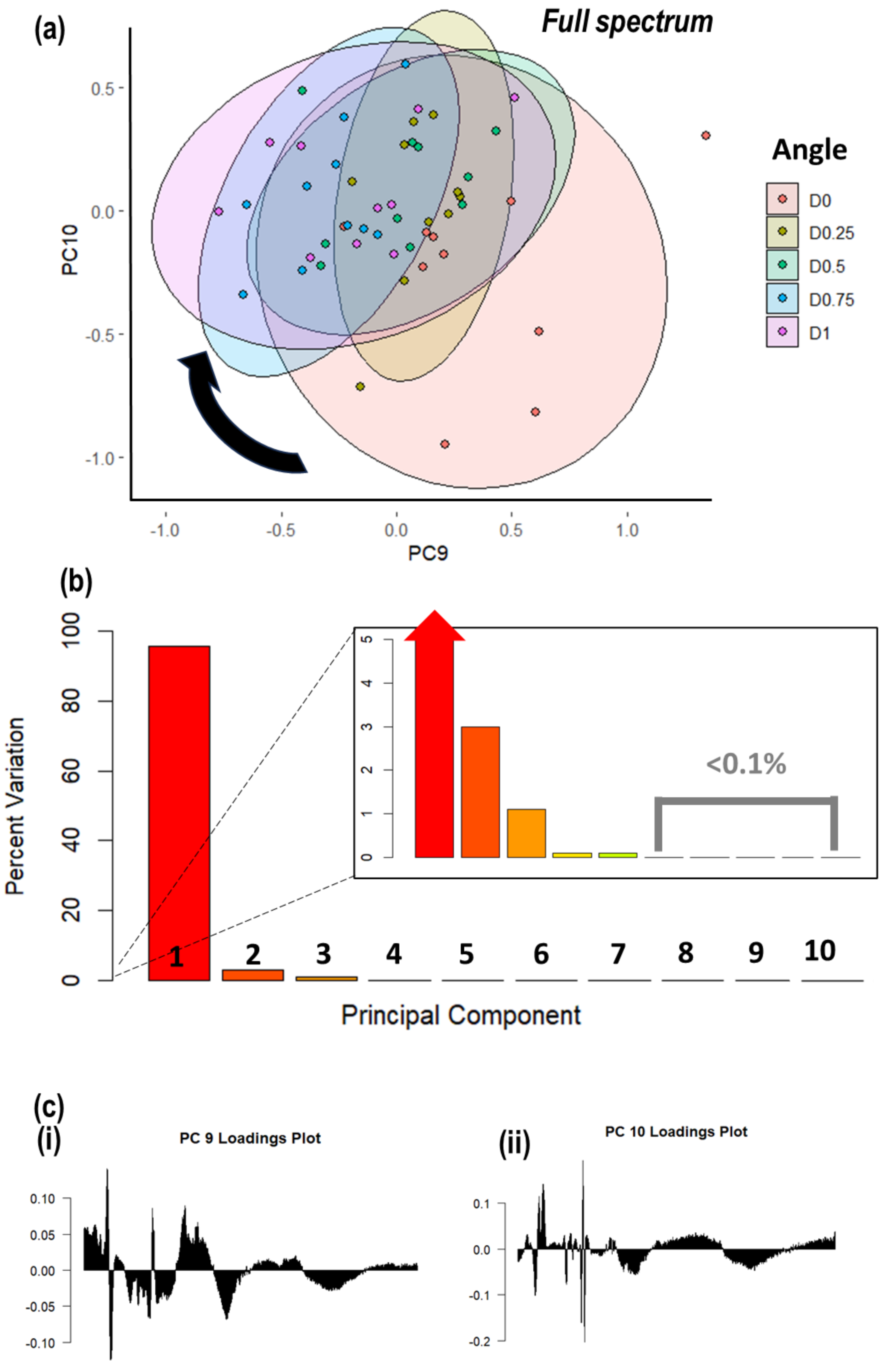

Figure S2. (a) PCA scores plot (PC 9 vs PC10) of spectra taken at small angles, 0°, 0.25°, 0.5°, 0.75°, and 1° rotation. N=10 spectra for each angle class. Bounded areas are confidence ellipses at 95%. (b) Corresponding scree plot showing variance explained for PCs 1-10, and (c) PCA loadings for (i) PC9 and (ii) PC10, demonstrating most important variables (wavelengths) incorporated into these PCs. Full spectrum = 400nm-1000nm.

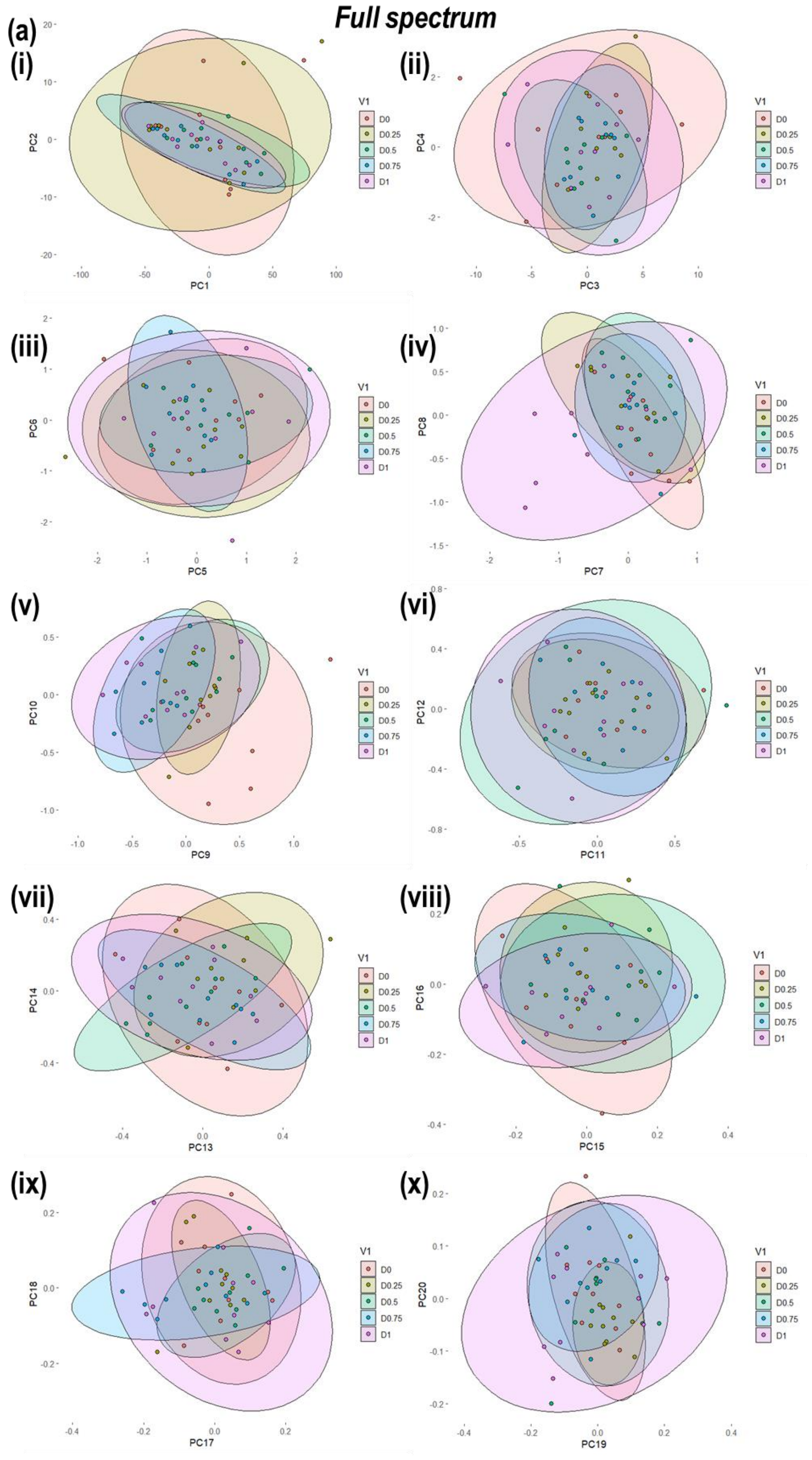

Figure S3. (a)(i)-(x) PCA scores plots up to PC20 of spectra taken at small angles, 0°, 0.25°, 0.5°, 0.75°, and 1° rotation for the full spectral range. N=10 spectra for each angle class. Bounded areas are confidence ellipses at 95%. Full spectrum = 400 nm-1000 nm.

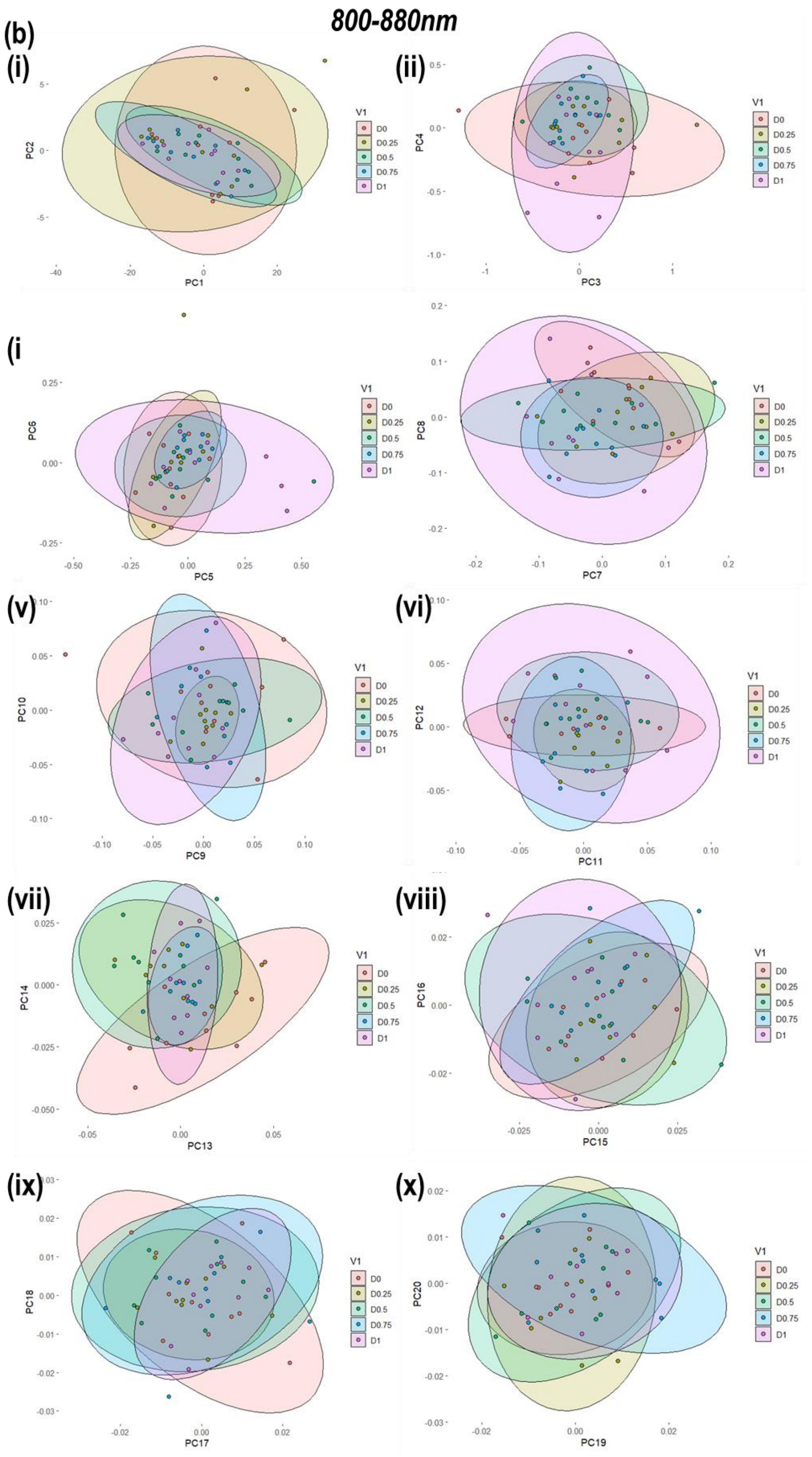

Figure S3 (cont.) (b)(i)-(x) PCA scores plots up to PC20 of spectra taken at small angles, 0°, 0.25°, 0.5°, 0.75°, and 1° rotation for truncated spectral range 800-880 nm. N=10 spectra for each angle class. Bounded areas are confidence ellipses at 95%.

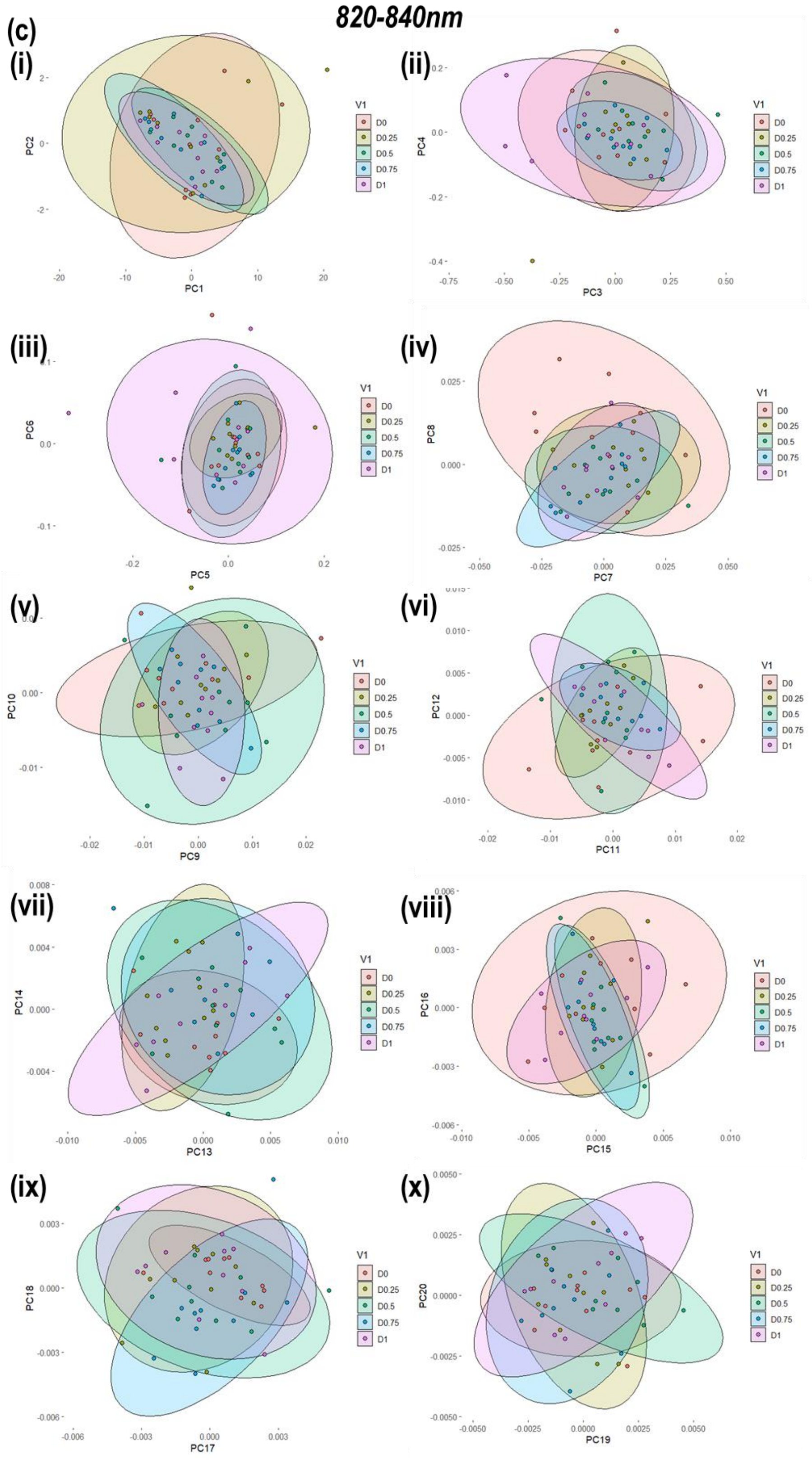

Figure S3. (cont.) (c)(i)-(x) PCA scores plots up to PC20 of spectra taken at small angles, 0°, 0.25°, 0.5°, 0.75°, and 1° rotation for the truncated spectral range (820-840 nm). N=10 spectra for each angle class. Bounded areas are confidence ellipses at 95%.

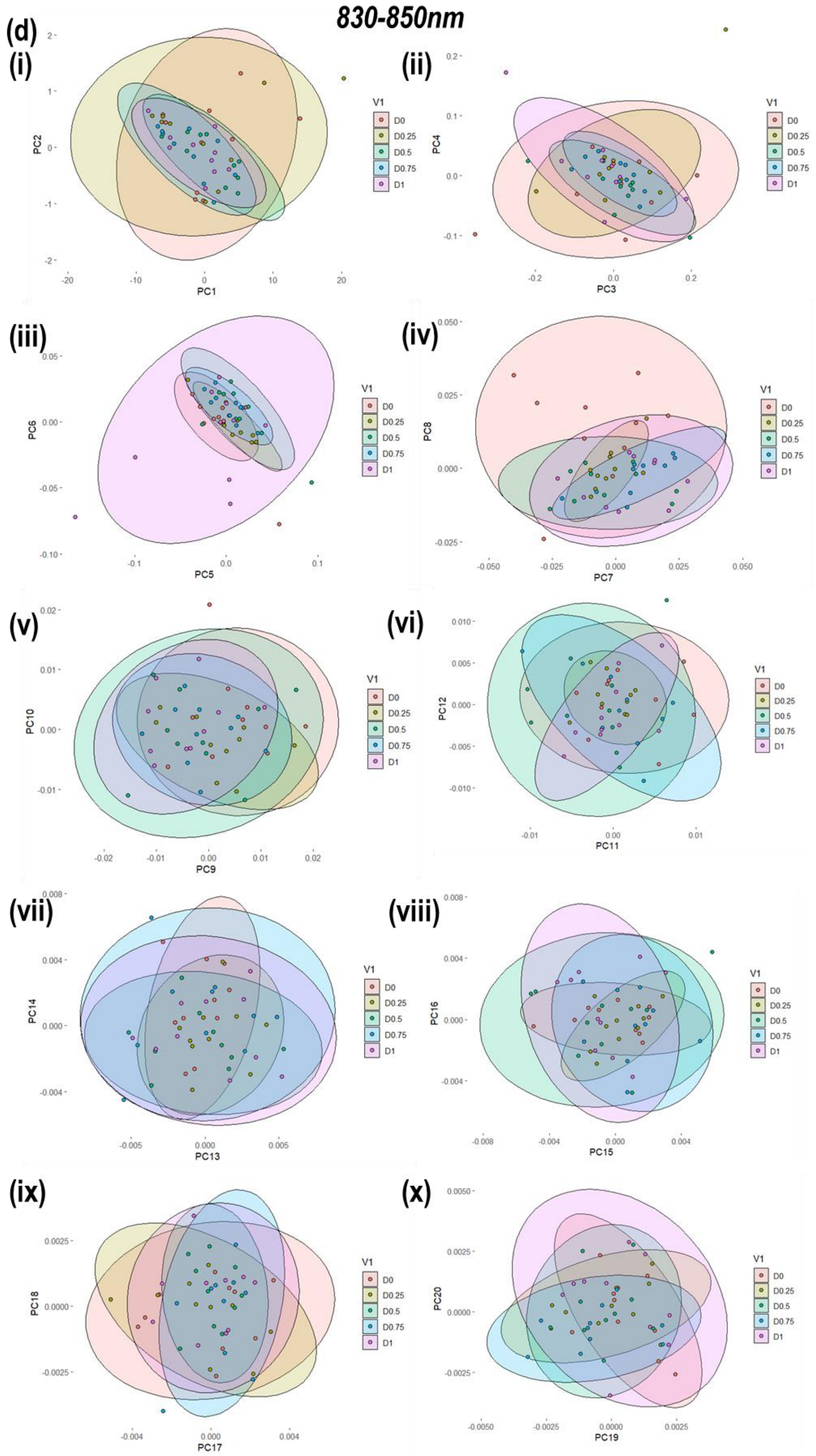

Figure S3. (cont.) (d)(i)-(x) PCA scores plots up to PC20 of spectra taken at small angles, 0°, 0.25°, 0.5°, 0.75°, and 1° rotation for the truncated spectral range (830-850 nm). N=10 spectra for each angle class. Bounded areas are confidence ellipses at 95%.

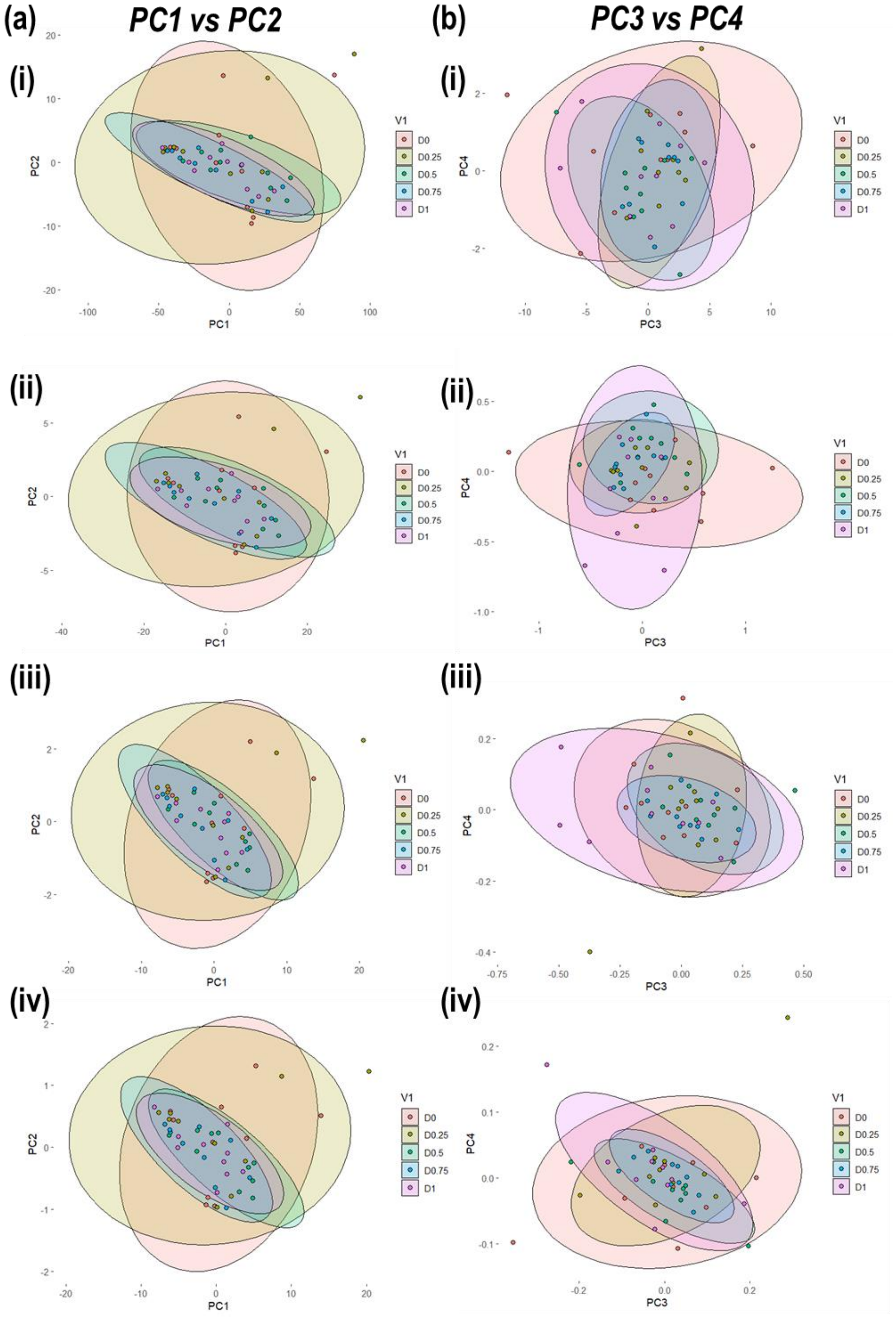

Figure S4. Replotted PCA score plots from Figure S3 for comparison of individual PCs from different spectral range selections. (a) For PC1 vs. PC2 for (i) full spectrum, (ii) 800-880 nm), (iii) 820-840 nm, (iv) 830-850 nm. (b) For PC3 vs. PC4 for (i) full spectrum, (ii) 800-880 nm), (iii) 820-840 nm, (iv) 830-850 nm.

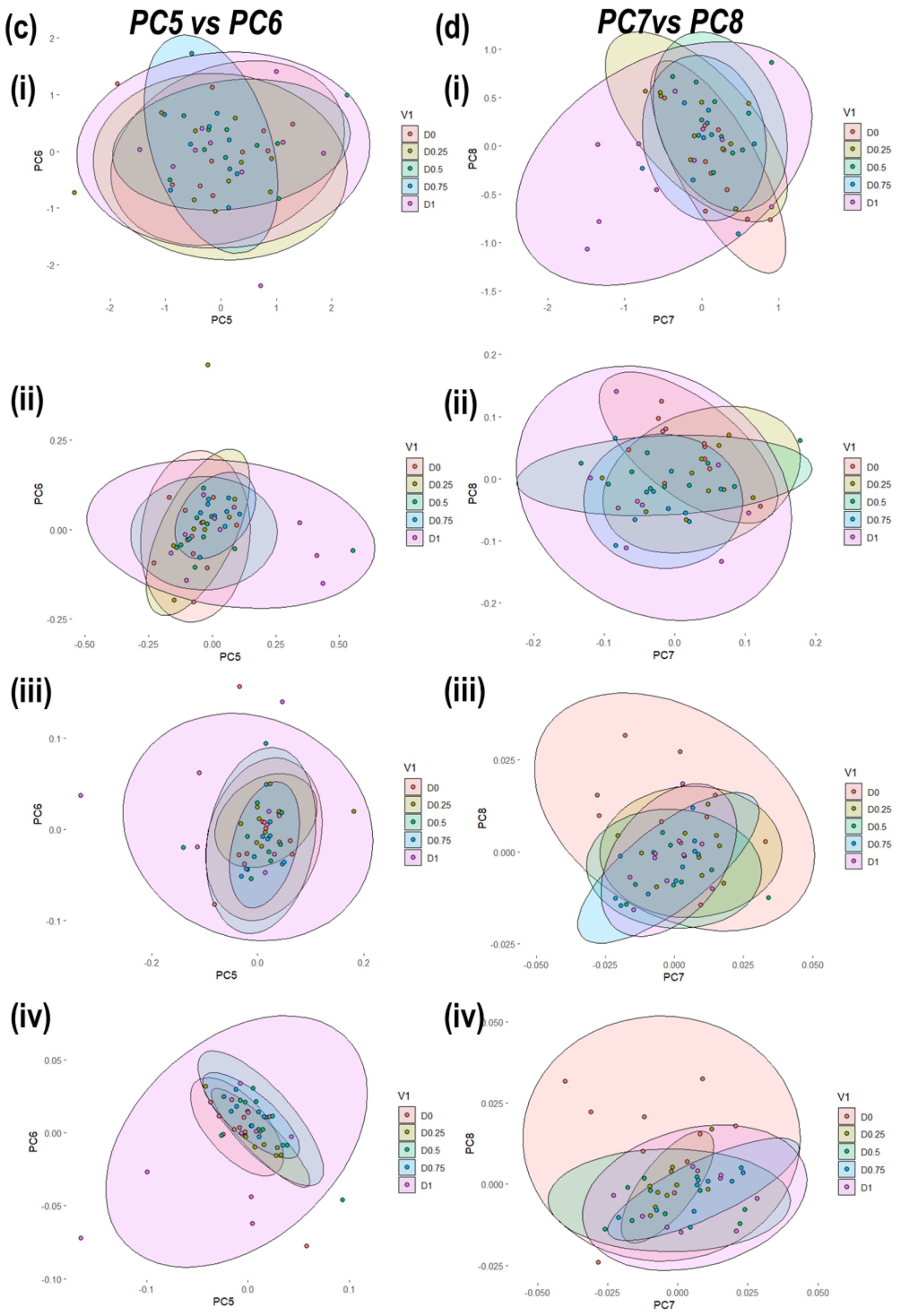

Figure S4. (cont.) Replotted PCA score plots from Figure S3 for comparison of individual PCs from different spectral range selections. (a) For PC4 vs. PC5 for (i) full spectrum, (ii) 800-880 nm), (iii) 820-840 nm, (iv) 830-850 nm. (b) For PC7 vs. PC8 for (i) full spectrum, (ii) 800-880 nm), (iii) 820-840 nm, (iv) 830-850 nm.

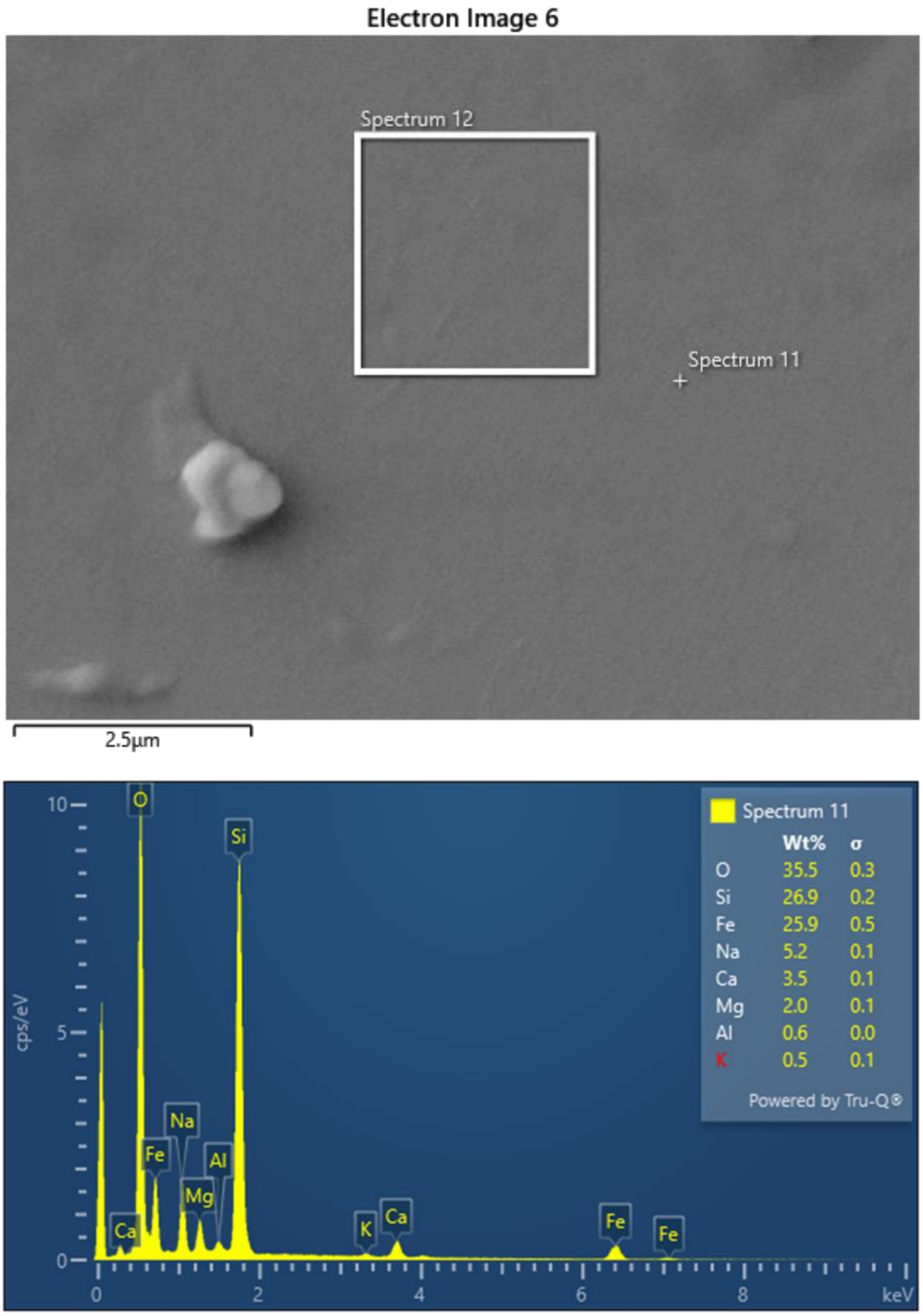

Figure S5. Compositional analysis of iron sample: (a) Scanning electron microscopy (SEM) micrograph and (b) corresponding electron dispersive x-ray spectroscopic (EDX) analysis.

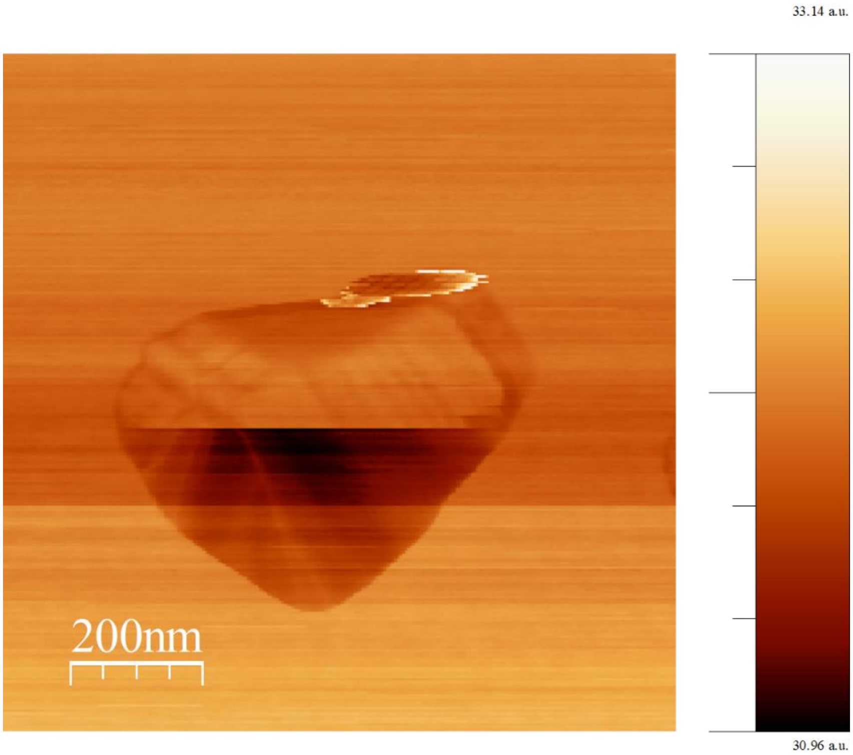

Figure S6. Scanning Thermal Microscopy image on Gold Nanodome showing temperature variation across one nanodome. Au thickness = 60 nm.

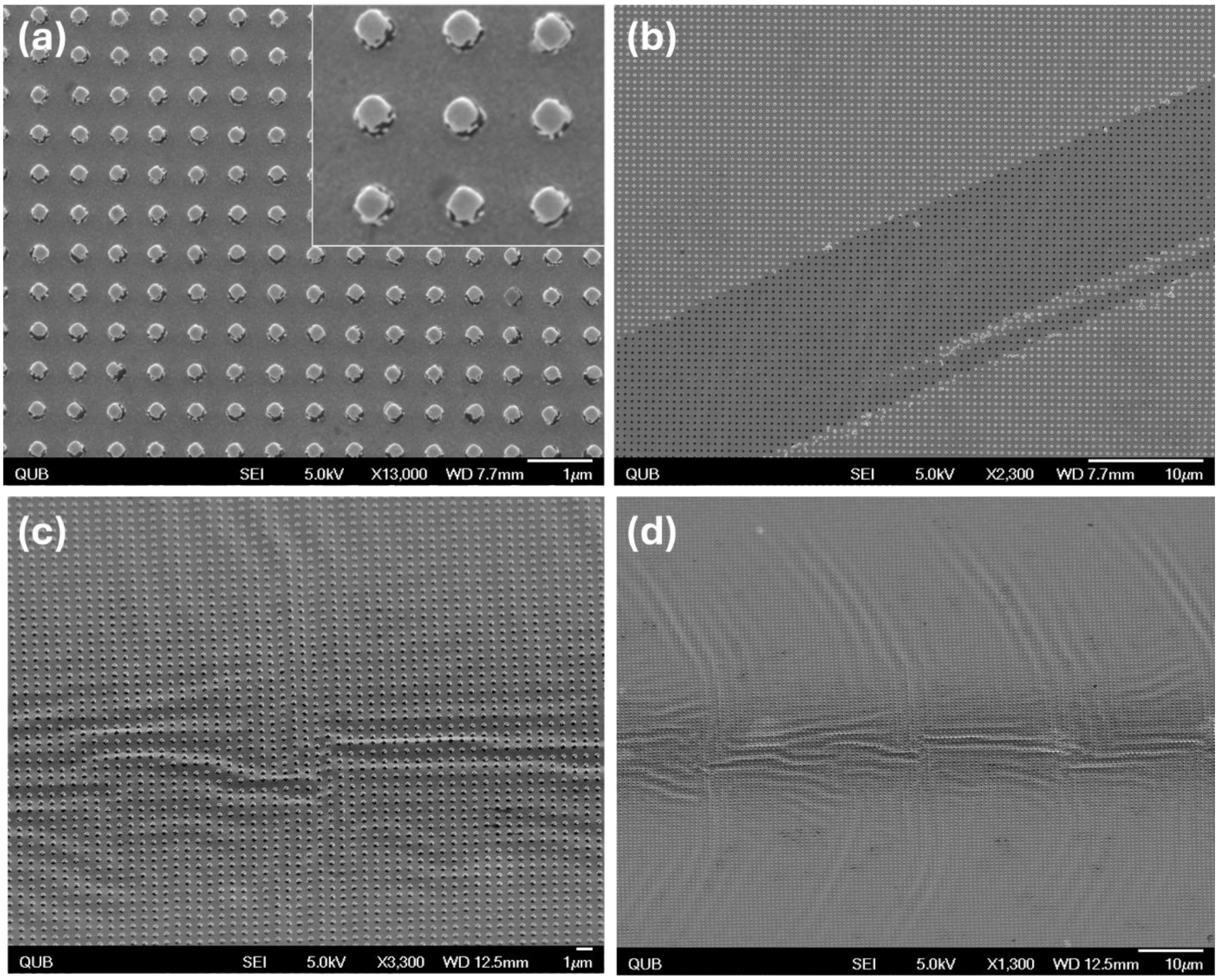

Figure S7. Possible laser damage in nanodome substrates. (a) Damage to individual nanostructures, INSET: Close-up on nanodomes, (b) larger-scale heating streak damage from laser. (c),(d) Warping defects zoom in in (c), and zoomed out in (d).

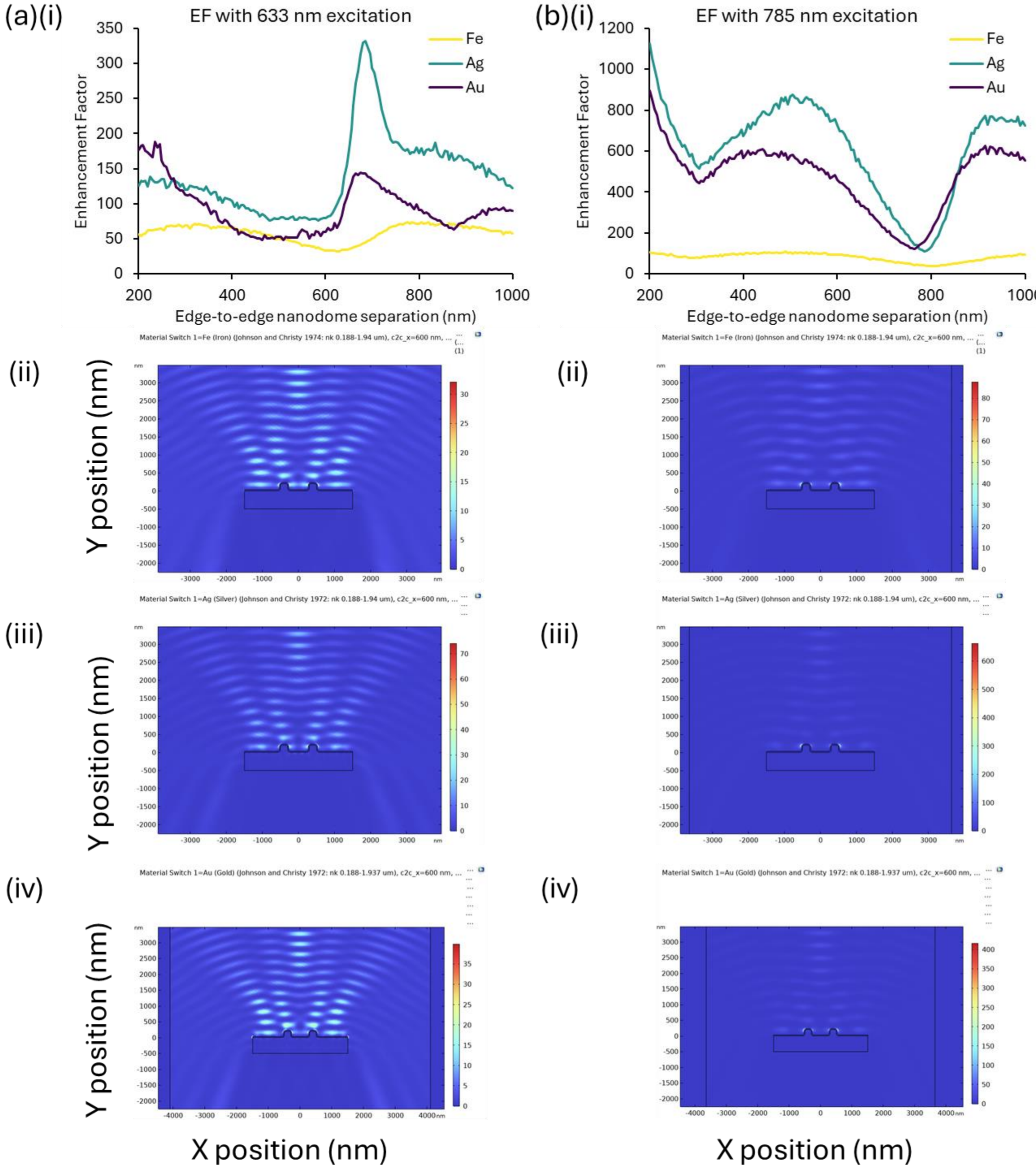

Figure S8. COMSOL models (2D) for two-dome with varying material and separation distance (dome edge to dome edge). for **(a)** 633 nm and **(b)** 785 nm excitation. **(a)(i)** SERS Enhancement Factor as a function of inter-dome spacing for iron, silver, and gold -coated nanodomes. **(a)(ii, iii, iv)** COMSOL plots for (i) iron, (ii) silver, and (iii) gold -coated nanodomes respectively. **(b)(ii, iii, iv)** Same but for 785 nm excitation. Metal thickness 30 nm all cases. Material data from Johnson and Christy (1972) (Ag & Au), & Johnson and Christy (1974) (Fe). Simulated EF is based on E^4 from the maximum electric field value on the nanodome base plane i.e. gold-air interface.

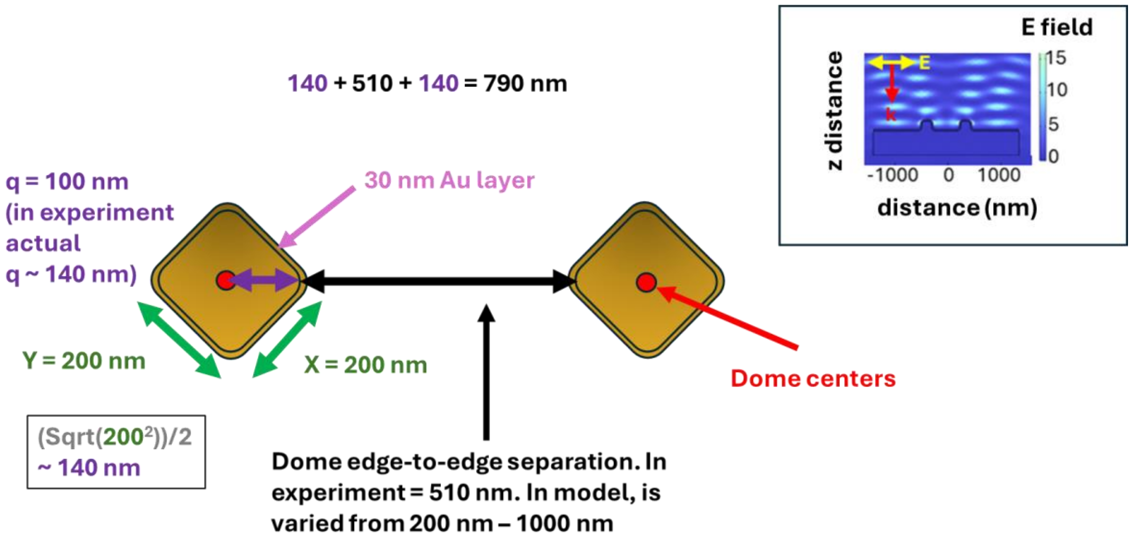

Figure S9. Explanative schematic for COMSOL models in Figure S8.
